# Supplementary material for: Intermittent BRAF inhibition in advanced BRAF mutated melanoma results of a phase II randomized trial
Source: Nat Commun. 2021 Dec 1;12:7008. doi: 10.1038/s41467-021-26572-6 (PMC8636498; doi:10.1038/s41467-021-26572-6)
Supplement: Supplementary file 1 — Supplementary Information [file 41467_2021_26572_MOESM1_ESM.pdf]

Supplementary Online Content

Maria Gonzalez-Cao; Clara Mayo de las Casas; Juana Oramas et al.
Intermittent BRAF inhibition in advanced BRAF mutated melanoma

This appendix has been provided by the authors to give readers additional information about their work.

INDEX

SUPPLEMENTARY METHODS 5

SUPPLEMENTARY FIGURE LEGENDS 5

Supplementary Figure 1. Treatment schedule 5

Supplementary Figure 2. Overall survival of BRAFV600 mutant melanoma patients by treatment arm 5

Supplementary Figure 3. A. Progression free survival according to the presence of BRAFV600 mutation in pretreatment cfDNA 5

Supplementary Figure 3. B. Overall survival according to the presence of BRAFV600 mutation in pretreatment cfDNA 5

Supplementary Figure 4. Overall survival according to the presence of BRAFV600 mutation in pretreatment cfDNA and treatment arm 5

Supplementary Figure 5. Progression free survival according to pretreatment LDH levels and the presence of BRAFV600 mutation in pretreatment cfDNA. 6

Supplementary Figure 6. Overall survival according to pretreatment LDH levels and the presence of BRAFV600 mutation in pretreatment cfDNA. 6

Supplementary Figure 7. Evolution along time of BRAFV600 mutation in cfDNA and tumor response by patient-treatment arm. 7

Supplementary Figure 8. Evolution along time of BRAFV600 mutation in cfDNA by treatment arm. 7

SUPPLEMENTARY TABLES 8

Supplementary Table 1. Patient Characteristics 8

Supplementary Table 2. Drug related adverse events (AEs) 10

Supplementary Table 3. Drug reductions and interruptions 11

Supplementary Table 4. Progression free survival analysis according to BRAFV600 cfDNA and treatment arm 12

Supplementary Table 5. Overall survival analysis according to BRAFV600 cfDNA and treatment arm 13

Supplementary Table 6. Univariate analysis of PFS – Sub-study population with basal BRAF cfDNA mITT population- 14

Supplementary Table 7. Multivariate analysis of PFS – Sub study population with basal BRAF cfDNA mITT (A) and PP population (B)- 15

Supplementary Table 8. Univariate analysis of OS – Sub study population with basal BRAF cfDNA mITT population- 17

Supplementary Table 9. Multivariate analysis of OS – Sub study population with basal BRAF cfDNA mITT (A) and PP population (B)- 19

Supplementary Table 10. Results of NGS analysis at Progression by treatment arm .. 20

SUPPLEMENTARY FIGURES 21

Supplementary Figure1 .... **¡Error! Marcador no definido.**

Supplementary Figure 2.. **¡Error! Marcador no definido.**
Supplementary Figure 3.. **¡Error! Marcador no definido.**
Supplementary Figure 4A.. **¡Error! Marcador no definido.**
Supplementary Figure 4B.. **¡Error! Marcador no definido.**
Supplementary Figure 5.. **¡Error! Marcador no definido.**
Supplementary Figure 6.. **¡Error! Marcador no definido.**
Supplementary Figure 7.. **¡Error! Marcador no definido.**
Supplementary Figure 8.. **¡Error! Marcador no definido.**
Supplementary Figure 9A... 46
Supplementary Figure 9B... 47

SUPPLEMENTARY METHODS

All analysis were performed by intended to treat population (iTTP). An exploratory analysis was performed in per protocol population (PP) (including those patients that received at least 4th cycle -those who did not progresses before starting a different schedule between arms- and those who treatment administration was according to protocol without mistakes in drug administration.

Main limitation of the study is a low number of patients included (n 70) due to a low recruitment rate.

Although there was no cross-over planned, once results were available, all participating centers were informed to discontinue treatment in patients in order to start standard therapy with BRAF plus MEK inhibitors, according to the approved schedule.

Analysis of BRAV600 mutations in cfDNA were performed in quadruplicate: two aliquots of serum and two aliquots of plasma per patient with similar results.

SUPPLEMENTARY FIGURE LEGENDS

Supplementary Figure 1. Treatment schedule

Supplementary Figure 2. Overall survival of BRAFV600 mutant melanoma patients by treatment arm (A continuous schedule, B intermittent schedule). Median overall survival (OS) was 23.59 months (95% CI 14.67- NA) in Group A- Continuous versus 27.53 months (95% CI 11.15 - NA) in Group B- Intermittent, and was statistically non-significant (p= 0.7293). *Overall survival was estimated by means of the Kaplan–Meier method and the nonparametric two-side log-rank test was applied for comparisons of groups.*

Supplementary Figure 3. A. Progression free survival according to the presence of BRAFV600 mutation in pretreatment cfDNA. There were significant differences in terms of median progression free survival according to BRAF detection in basal cfDNA with a median PFS not reached (95% CI 2.76, NR) in patients without BRAF detection (preBRAF-) versus 14.67 months (95% CI 8.52, 23.59) in preBRAF+, p=0.0518**.** *Progression free survival was estimated by means of the Kaplan–Meier method and the nonparametric two-side log-rank test was applied for comparisons of groups.*

Supplementary Figure 3. B. Overall survival according to the presence of BRAFV600 mutation in pretreatment cfDNA. There were significant differences in terms of median survival according to BRAF detection in basal cfDNA with a median OS not reached (95% CI 32.63, NR) in patients preBRAF- versus 8.26 months in preBRAF+ (95% CI 5.20, 18.88), p=0.0024**.** *Overall survival was estimated by means of the Kaplan–Meier method and the nonparametric two-side log-rank test was applied for comparisons of groups.*

Supplementary Figure 4. Overall survival according to the presence of BRAFV600 mutation in pretreatment cfDNA and treatment arm. There were significant

differences in terms of median survival according to BRAF detection in basal cfDNA in each treatment arm. In the continuous arm, median OS was 21.6 months (95% CI 5.4,NR) in preBRAF+, and NR for preBRAF- (95% CI 3.5,NR). For the intermittent arm, median OS was 10 months (95% CI 0.4-27.5) for preBRAF+, and NR for preBRAF- (95%CI 15.9, NR) (p=0.009). *Overall survival was estimated by means of the Kaplan–Meier method and the nonparametric two-side log-rank test was applied for comparisons of groups.*

Footnote:

G1: Continuous arm and basal BRAF Positive in cfDNA, G2: Intermittent arm and basal BRAF Positive in cfDNA, G3: Continuous arm and basal BRAF Negative in cfDNA, G4: Intermittent arm and basal BRAF Negative in cfDNA

Supplementary Figure 5. Progression free survival according to pretreatment LDH levels and the presence of BRAFV600 mutation in pretreatment cfDNA. There were significant differences in terms of median progression free survival according to BRAF detection in basal cfDNA in patients with normal LDH levels. There was only one patient with LDH Elevated and basal BRAF Negative (id=0105) . For this reason, the group Elevated - does not appear in the survival analysis. Patients with high LDH levels had a median PFS of 7.9 months (95% CI 2.5,13.6), while patients with normal LDH levels and BRAF detection in pretreatment cfDNA had a median PFS of 8.2 months (95% CI 4.3,NR) and patients with normal LDH levels and no BRAF detection in cfDNA had NR months (95% CI 5.3,NR) (p=0.0112). *Progression free survival was estimated by means of the Kaplan–Meier method and the nonparametric two-side log-rank test was applied for comparisons of groups.*

Footnote: Normal -: Normal LDH levels and BRAF negative in cfDNA pretreatment; Normal +: Normal LDH levels and BRAF positive in cfDNA pretreatment; Elevated +: High LDH levels and BRAF positive in cfDNA pretreatment

Supplementary Figure 6. Overall survival according to pretreatment LDH levels and the presence of BRAFV600 mutation in pretreatment cfDNA. There were significant differences in terms of median overall survival according to BRAF detection on basal cfDNA in patients with normal LDH levels. There was only one patient withLDH elevated and basal BRAF Negative (id=0105). For this reason, the group Elevated - does not appear in the survival analysis. Patients with high LDH levels had a median OS of 12.4 months (95% CI 5.4,27.5), while for patients with normal LDH levels and BRAF detection in pretreatment cfDNA, median OS was 23 months (95% CI 6.9,NR), and for patients with normal LDH levels and no BRAF detection in cfDNA, it was NR months (95% CI 32.6,NR) (p=0.0020). *Overall survival was estimated by means of the Kaplan–Meier method and the nonparametric two-side log-rank test was applied for comparisons of groups.*

Footnote: Normal -: Normal LDH levels and BRAF negative in cfDNA pretreatment; Normal +: Normal LDH levels and BRAF positive in cfDNA pretreatment; Elevated +: High LDH levels and BRAF positive in cfDNA pretreatment

Supplementary Figure 7. Evolution along time of BRAFV600 mutation in cfDNA and tumor response by patient-treatment arm. A (continuous) and B (intermittent). In this section, a graphic of the evolution of BRAF cfDNA in plasma and serum over time and the tumor response is presented for some patients The y axis will correspond to the measures of cfDNA using pg/uL and % of mutated allele . The x axis will show the time, in weeks, from the treatment start date. *Footnote: TRT: Treatment arm*

Supplementary Figure 8. Evolution along time of BRAFV600 mutation in cfDNA by treatment arm. **A** Continuous arm A; **B** Intermittent arm B. In this section, a graphic of the evolution of BRAF cfDNA in blood over time and the tumor response is presented for all patients. The y axis will correspond to the measures of cfDNA using % of mutated allele . The x axis will show the time, in weeks, from the treatment start date.

SUPPLEMENTARY TABLES

Characteristics	Arm A (n 35)	Arm B (n 35)	Global (n 70)
**Age – y**			
Median (range)	58 (49-69)	56 (29-85)	57 (29-85)
**Sex – n (%)**			
Women	11 (31)	22 (63)	33 (47)
**ECOG PS – n (%)**			
0	19 (54)	21 (60)	40 (57)
1	16 (46)	14 (40)	30 (43)
**Primary melanoma – n (%)**			
Cutaneous	29 (83)	28 (79)	57 (81)
Mucosal	0	1 (3)	1 (1)
Acral	1 (3)	2 (6)	3 (4)
Unknown primary	5 (14)	4 (12)	9 (13)
**Stage – n (%)**			
IIIc	0	1 (3)	1 (1)
M1a	8 (23)	6 (17)	14 (20)
M1b	11 (31)	8 (23)	18 (26)
M1c	16 (46)	20 (57)	36 (51)
**LDH – n (%)**			
**Normal**	19 (54)	20 (57)	39 (56)
**Elevated (≤2x ULN)**	10 (29)	9 (26)	19 (27)
**Elevated (>2x ULN)**	6 (17)	6 (17)	12 (17)
**Number of metastatic sites- n (%)**			
0	0	1 (3)	1 (1)
1	12 (34.3)	6 (17.1)	18 (25.7)
2	10 (29)	10 (29)	10 (29)
>2	13 (37)	18 (51)	31 (44)
**Prior adjuvant therapy- n (%)**			
Interferon	9 (26)	18 (51)	31 (44)
Nivolumab	9 (26)	7 (20)	16 (22)
Ipilimumab	1 (3)	0	1 (1)

Supplementary Table 1. Patient Characteristics

Footnote: Arm A: continuous schedule; Arm B: intermittent schedule; PS: performance status

	Arm A (N=35)								Arm B (N=35)							
	Grade 1		Grade 2		Grade 3		Grade 4		Grade 1		Grade 2		Grade 3		Grade 4	
**System Organ Class/ Preferred Term**	n(%)		n(%)		n(%)		n(%)		n(%)		n(%)		n(%)		n(%)	
**Overall**	**1**	**(2.85)**	**15**	**(42.85)**	**13**	**(37.14)**	**2**	**(5.71)**	**3**	**(8.57)**	**5**	**(14.28)**	**12**	**(34.28)**	**2**	**(5.71)**
**Blood and lymphatic system disorders**	**2**	**(5.71)**	**2**	**(5.71)**	**1**	**(2.85)**	**0**	**(0.00)**	**1**	**(2.85)**	**0**	**(0.00)**	**0**	**(0.00)**	**0**	**(0.00)**
Anaemia	2	(5.71)	2	(5.71)	0	(0.00)	0	(0.00)	0	(0.00)	0	(0.00)	0	(0.00)	0	(0.00)
Leukopenia	1	(2.85)	0	(0.00)	0	(0.00)	0	(0.00)	1	(2.85)	0	(0.00)	0	(0.00)	0	(0.00)
Lymphopenia	0	(0.00)	0	(0.00)	1	(2.85)	0	(0.00)	0	(0.00)	0	(0.00)	0	(0.00)	0	(0.00)
Neutropenia	2	(5.71)	0	(0.00)	0	(0.00)	0	(0.00)	0	(0.00)	0	(0.00)	0	(0.00)	0	(0.00)
**Eye disorders**	**4**	**(11.42)**	**11**	**(31.42)**	**1**	**(2.85)**	**0**	**(0.00)**	**4**	**(11.42)**	**2**	**(5.71)**	**0**	**(0.00)**	**0**	**(0.00)**
Chorioretinopathy	0	(0.00)	1	(2.85)	1	(2.85)	0	(0.00)	0	(0.00)	0	(0.00)	0	(0.00)	0	(0.00)
Dry eye	0	(0.00)	0	(0.00)	0	(0.00)	0	(0.00)	1	(2.85)	0	(0.00)	0	(0.00)	0	(0.00)
Iridocyclitis	0	(0.00)	1	(2.85)	0	(0.00)	0	(0.00)	0	(0.00)	0	(0.00)	0	(0.00)	0	(0.00)
Maculopathy	0	(0.00)	1	(2.85)	0	(0.00)	0	(0.00)	0	(0.00)	1	(2.85)	0	(0.00)	0	(0.00)
Retinal detachment	1	(2.85)	4	(9.43)	0	(0.00)	0	(0.00)	0	(0.00)	1	(2.85)	0	(0.00)	0	(0.00)
Serous retinopathy	0	(0.00)	1	(2.85)	0	(0.00)	0	(0.00)	0	(0.00)	0	(0.00)	0	(0.00)	0	(0.00)
Uveitis	0	(0.00)	2	(5.71)	0	(0.00)	0	(0.00)	0	(0.00)	0	(0.00)	0	(0.00)	0	(0.00)
Vision blurred	1	(2.85)	0	(0.00)	0	(0.00)	0	(0.00)	2	(5.71)	0	(0.00)	0	(0.00)	0	(0.00)
Visual impairment	1	(2.85)	1	(2.85)	0	(0.00)	0	(0.00)	0	(0.00)	0	(0.00)	0	(0.00)	0	(0.00)
Vitritis	0	(0.00)	1	(2.85)	0	(0.00)	0	(0.00)	0	(0.00)	0	(0.00)	0	(0.00)	0	(0.00)
**Gastrointestinal disorders**	**9**	**(25.71)**	**7**	**(20.00)**	**1**	**(2.85)**	**0**	**(0.00)**	**6**	**(17.14)**	**4**	**(11.42)**	**3**	**(8.57)**	**0**	**(0.00)**
Abdominal pain	2	(5.71)	0	(0.00)	0	(0.00)	0	(0.00)	0	(0.00)	0	(0.00)	0	(0.00)	0	(0.00)
Cheilitis	1	(2.85)	0	(0.00)	0	(0.00)	0	(0.00)	0	(0.00)	0	(0.00)	0	(0.00)	0	(0.00)
Constipation	1	(2.85)	0	(0.00)	0	(0.00)	0	(0.00)	0	(0.00)	0	(0.00)	0	(0.00)	0	(0.00)
Diarrhoea	6	(17.14)	5	(14.28)	0	(0.00)	0	(0.00)	4	(11.42)	3	(8.57)	3	(8.57)	0	(0.00)
Gastrooesophageal reflux disease	0	(0.00)	0	(0.00)	0	(0.00)	0	(0.00)	1	(2.85)	0	(0.00)	0	(0.00)	0	(0.00)
Gingival bleeding	0	(0.00)	1	(2.85)	0	(0.00)	0	(0.00)	0	(0.00)	0	(0.00)	0	(0.00)	0	(0.00)
Nausea	4	(11.42)	2	(5.71)	0	(0.00)	0	(0.00)	2	(5.71)	2	(5.71)	0	(0.00)	0	(0.00)
Odynophagia	1	(2.85)	0	(0.00)	0	(0.00)	0	(0.00)	0	(0.00)	0	(0.00)	0	(0.00)	0	(0.00)
Stomatitis	2	(5.40)	1	(2.85)	1	(2.85)	0	(0.00)	0	(0.00)	0	(0.00)	0	(0.00)	0	(0.00)
Vomiting	5	(14.28)	1	(2.85)	0	(0.00)	0	(0.00)	0	(0.00)	2	(5.71)	2	(5.71)	0	(0.00)
**General disorders**	**5**	**(14.28)**	**9**	**(25.71)**	**3**	**(8.57)**	**0**	**(0.00)**	**8**	**(22.85)**	**3**	**(8.57)**	**3**	**(8.57)**	**0**	**(0.00)**
Asthenia	5	(14.27)	8	(22.85)	2	(5.71)	0	(0.00)	6	(17.14)	4	(11.42)	3	(8.57)	0	(0.00)
Face oedema	0	(0.00)	0	(0.00)	1	(2.85)	0	(0.00)	1	(2.85)	0	(0.00)	0	(0.00)	0	(0.00)
Oedema peripheral	1	(2.85)	0	(0.00)	0	(0.00)	0	(0.00)	2	(5.71)	0	(0.00)	0	(0.00)	0	(0.00)
Pyrexia	3	(8.57)	0	(0.00)	0	(0.00)	0	(0.00)	5	(14.28)	0	(0.00)	0	(0.00)	0	(0.00)
Xerosis	3	(8.57)	0	(0.00)	0	(0.00)	0	(0.00)	0	(0.00)	0	(0.00)	0	(0.00)	0	(0.00)
Hepatotoxicity	0	(0.00)	1	(2.85)	0	(0.00)	0	(0.00)	0	(0.00)	0	(0.00)	0	(0.00)	0	(0.00)
**Infections**	**3**	**(8.57)**	**1**	**(2.85)**	**0**	**(0.00)**	**0**	**(0.00)**	**1**	**(2.85)**	**1**	**(2.85)**	**0**	**(0.00)**	**0**	**(0.00)**
Candida infection	1	(2.85)	0	(0.00)	0	(0.00)	0	(0.00)	0	(0.00)	0	(0.00)	0	(0.00)	0	(0.00)
Conjunctivitis	2	(5.71)	0	(0.00)	0	(0.00)	0	(0.00)	0	(0.00)	0	(0.00)	0	(0.00)	0	(0.00)
Folliculitis	0	(0.00)	0	(0.00)	0	(0.00)	0	(0.00)	1	(2.85)	1	(2.85)	0	(0.00)	0	(0.00)
Gingivitis	0	(0.00)	1	(2.85)	0	(0.00)	0	(0.00)	0	(0.00)	0	(0.00)	0	(0.00)	0	(0.00)
**Analytical alterations**	**1**	**(2.85)**	**3**	**(8.57)**	**2**	**(5.71)**	**0**	**(0.00)**	**0**	**(0.00)**	**0**	**(0.00)**	**1**	**(2.85)**	**0**	**(0.00)**
Alanine aminotransferase increased	3	(8.57)	0	(0.00)	0	(0.00)	0	(0.00)	0	(0.00)	0	(0.00)	0	(0.00)	0	(0.00)
Amylase increased	1	(2.85)	0	(0.00)	0	(0.00)	0	(0.00)	0	(0.00)	0	(0.00)	0	(0.00)	0	(0.00)
Aspartate aminotransferase increased	2	(5.71)	0	(0.00)	0	(0.00)	0	(0.00)	0	(0.00)	0	(0.00)	0	(0.00)	0	(0.00)
Blood alkaline phosphatase increased	1	(2.85)	1	(2.85)	1	(2.85)	0	(0.00)	0	(0.00)	0	(0.00)	0	(0.00)	0	(0.00)
Blood bilirubin increased	1	(2.85)	0	(0.00)	0	(0.00)	0	(0.00)	0	(0.00)	0	(0.00)	0	(0.00)	0	(0.00)
Blood calcium increased	1	(2.85)	0	(0.00)	0	(0.00)	0	(0.00)	0	(0.00)	0	(0.00)	0	(0.00)	0	(0.00)
Blood cholesterol increased	2	(5.71)	0	(0.00)	0	(0.00)	0	(0.00)	0	(0.00)	0	(0.00)	0	(0.00)	0	(0.00)
Blood creatine phosphokinase increased	1	(2.85)	2	(5.71)	0	(0.00)	0	(0.00)	0	(0.00)	0	(0.00)	1	(2.85)	0	(0.00)
Blood creatinine increased	0	(0.00)	1	(2.85)	0	(0.00)	0	(0.00)	0	(0.00)	0	(0.00)	0	(0.00)	0	(0.00)
Blood lactate dehydrogenase increased	1	(2.85)	0	(0.00)	0	(0.00)	0	(0.00)	0	(0.00)	0	(0.00)	0	(0.00)	0	(0.00)
Electrocardiogram QT prolonged	1	(2.85)	0	(0.00)	0	(0.00)	0	(0.00)	0	(0.00)	0	(0.00)	0	(0.00)	0	(0.00)
Lipase increased	0	(0.00)	0	(0.00)	1	(2.85)	0	(0.00)	0	(0.00)	0	(0.00)	0	(0.00)	0	(0.00)
**Metabolism and nutrition disorders**	**3**	**(8.57)**	**2**	**(5.71)**	**0**	**(0.00)**	**0**	**(0.00)**	**4**	**(11.42)**	**1**	**(2.85)**	**0**	**(0.00)**	**0**	**(0.00)**
Decreased appetite	2	(5.71)	2	(5.71)	0	(0.00)	0	(0.00)	3	(8.57)	1	(2.85)	0	(0.00)	0	(0.00)
Hypokalaemia	0	(0.00)	0	(0.00)	0	(0.00)	0	(0.00)	1	(2.85)	0	(0.00)	0	(0.00)	0	(0.00)
Hyposideraemia	1	(2.85)	1	(2.85)	0	(0.00)	0	(0.00)	0	(0.00)	0	(0.00)	0	(0.00)	0	(0.00)
**Musculoskeletal and connective tissue disorders**	**4**	**(11.42)**	**3**	**(8.57)**	**2**	**(5.71)**	**0**	**(0.00)**	**5**	**(14.28)**	**2**	**(5.71)**	**1**	**(2.85)**	**0**	**(0.00)**
Arthralgia	3	(8.57)	1	(2.85)	2	(5.71)	0	(0.00)	5	(14.28)	2	(5.71)	0	(0.00)	0	(0.00)
Muscular weakness	0	(0.00)	0	(0.00)	0	(0.00)	0	(0.00)	0	(0.00)	0	(0.00)	1	(2.85)	0	(0.00)
Musculoskeletal pain	1	(2.85)	1	(2.85)	0	(0.00)	0	(0.00)	1	(2.85)	1	(2.85)	0	(0.00)	0	(0.00)
Myalgia	1	(2.85)	1	(2.85)	0	(0.00)	0	(0.00)	0	(0.00)	0	(0.00)	0	(0.00)	0	(0.00)
Polyarthritis	0	(0.00)	1	(2.85)	0	(0.00)	0	(0.00)	0	(0.00)	0	(0.00)	0	(0.00)	0	(0.00)
Tenosynovitis	1	(2.85)	0	(0.00)	0	(0.00)	0	(0.00)	0	(0.00)	0	(0.00)	0	(0.00)	0	(0.00)
**Neoplasms benign, malignant and unspecified (incl cysts and polyps)**	**1**	**(2.85)**	**0**	**(0.00)**	**3**	**(8.57)**	**0**	**(0.00)**	**0**	**(0.00)**	**0**	**(0.00)**	**0**	**(0.00)**	**0**	**(0.00)**
Keratoacanthoma	0	(0.00)	0	(0.00)	2	(5.71)	0	(0.00)	0	(0.00)	0	(0.00)	0	(0.00)	0	(0.00)
Skin papilloma	1	(2.85)	0	(0.00)	0	(0.00)	0	(0.00)	0	(0.00)	0	(0.00)	0	(0.00)	0	(0.00)
Squamous cell carcinoma	0	(0.00)	0	(0.00)	1	(2.85)	0	(0.00)	0	(0.00)	0	(0.00)	0	(0.00)	0	(0.00)
**Nervous system disorders**	**2**	**(5.71)**	**1**	**(2.85)**	**0**	**(0.00)**	**0**	**(0.00)**	**6**	**(17.14)**	**0**	**(0.00)**	**0**	**(0.00)**	**0**	**(0.00)**
Dizziness	0	(0.00)	0	(0.00)	0	(0.00)	0	(0.00)	2	(5.71)	0	(0.00)	0	(0.00)	0	(0.00)
Headache	1	(2.85)	0	(0.00)	0	(0.00)	0	(0.00)	4	(11.42)	0	(0.00)	0	(0.00)	0	(0.00)
Neuropathy peripheral	0	(0.00)	1	(2.85)	0	(0.00)	0	(0.00)	0	(0.00)	0	(0.00)	0	(0.00)	0	(0.00)
**Renal and urinary disorders**	**0**	**(0.00)**	**1**	**(2.85)**	**0**	**(0.00)**	**0**	**(0.00)**	**0**	**(0.00)**	**0**	**(0.00)**	**0**	**(0.00)**	**0**	**(0.00)**
Proteinuria	0	(0.00)	1	(2.85)	0	(0.00)	0	(0.00)	0	(0.00)	0	(0.00)	0	(0.00)	0	(0.00)
Renal failure	1	(2.85)	0	(0.00)	0	(0.00)	0	(0.00)	0	(0.00)	0	(0.00)	0	(0.00)	0	(0.00)
**Skin and subcutaneous tissue disorders**	**7**	**(20.00)**	**12**	**(34.28)**	**6**	**(17.14)**	**2**	**(5.71)**	**7**	**(20.00)**	**3**	**(8.57)**	**6**	**(17.14)**	**2**	**(5.71)**

Acne	2	(5.71)	0	(0.00)	1	(2.85)	0	(0.00)	0	(0.00)	0	(0.00)	0	(0.00)	0	(0.00)
Actinic keratosis	1	(2.85)	0	(0.00)	0	(0.00)	0	(0.00)	0	(0.00)	0	(0.00)	0	(0.00)	0	(0.00)
Alopecia	3	(8.57)	1	(2.85)	0	(0.00)	0	(0.00)	2	(5.71)	1	(2.85)	0	(0.00)	0	(0.00)
Angioedema	0	(0.00)	0	(0.00)	1	(2.85)	0	(0.00)	0	(0.00)	0	(0.00)	0	(0.00)	0	(0.00)
Dermatitis acneiform	0	(0.00)	0	(0.00)	0	(0.00)	0	(0.00)	2	(5.71)	0	(0.00)	1	(2.85)	0	(0.00)
Dermatitis allergic	0	(0.00)	1	(2.85)	0	(0.00)	0	(0.00)	0	(0.00)	0	(0.00)	0	(0.00)	0	(0.00)
Dermatitis atopic	0	(0.00)	1	(2.85)	0	(0.00)	0	(0.00)	0	(0.00)	0	(0.00)	0	(0.00)	0	(0.00)
Dermatitis exfoliative generalised	0	(0.00)	0	(0.00)	0	(0.00)	0	(0.00)	0	(0.00)	0	(0.00)	0	(0.00)	1	(2.85)
Drug reaction with eosinophilia and systemic symptoms	0	(0.00)	0	(0.00)	1	(2.85)	0	(0.00)	0	(0.00)	0	(0.00)	0	(0.00)	0	(0.00)
Dry skin	3	(8.57)	0	(0.00)	1	(2.85)	0	(0.00)	1	(2.85)	0	(0.00)	0	(0.00)	0	(0.00)
Eczema asteatotic	0	(0.00)	0	(0.00)	0	(0.00)	0	(0.00)	1	(2.85)	0	(0.00)	0	(0.00)	0	(0.00)
Erythema	1	(2.85)	3	(8.57)	0	(0.00)	0	(0.00)	1	(2.85)	0	(0.00)	0	(0.00)	0	(0.00)
Erythema multiforme	0	(0.00)	0	(0.00)	0	(0.00)	1	(2.85)	0	(0.00)	0	(0.00)	0	(0.00)	0	(0.00)
Erythema nodosum	0	(0.00)	0	(0.00)	0	(0.00)	0	(0.00)	0	(0.00)	1	(2.85)	0	(0.00)	0	(0.00)
Hyperkeratosis	1	(2.85)	0	(0.00)	0	(0.00)	0	(0.00)	0	(0.00)	0	(0.00)	0	(0.00)	0	(0.00)
Nail dystrophy	1	(2.85)	0	(0.00)	0	(0.00)	0	(0.00)	0	(0.00)	0	(0.00)	0	(0.00)	0	(0.00)
Palmar-plantar erythrodysaesthesia syndrome	1	(2.85)	0	(0.00)	0	(0.00)	0	(0.00)	0	(0.00)	0	(0.00)	0	(0.00)	0	(0.00)
Palmoplantar keratoderma	0	(0.00)	1	(2.85)	0	(0.00)	0	(0.00)	0	(0.00)	0	(0.00)	0	(0.00)	0	(0.00)
Panniculitis	1	(2.85)	3	(8.57)	0	(0.00)	0	(0.00)	0	(0.00)	0	(0.00)	0	(0.00)	0	(0.00)
Photosensitivity reaction	5	(14.27)	4	(11.42)	0	(0.00)	0	(0.00)	1	(2.85)	0	(0.00)	1	(2.85)	0	(0.00)
Pruritus	7	(20.00)	2	(5.71)	1	(2.85)	0	(0.00)	2	(5.71)	2	(5.71)	0	(0.00)	0	(0.00)
Rash	5	(14.28)	1	(2.85)	1	(2.85)	0	(0.00)	4	(11.42)	2	(5.71)	2	(5.71)	0	(0.00)
Rash erythematous	1	(2.85)	0	(0.00)	0	(0.00)	0	(0.00)	0	(0.00)	0	(0.00)	0	(0.00)	0	(0.00)
Rash maculo-papular	0	(0.00)	1	(2.85)	0	(0.00)	1	(2.85)	0	(0.00)	0	(0.00)	3	(8.57)	0	(0.00)
Skin exfoliation	0	(0.00)	1	(2.85)	0	(0.00)	0	(0.00)	0	(0.00)	1	(2.85)	0	(0.00)	0	(0.00)
Skin fissures	0	(0.00)	0	(0.00)	0	(0.00)	0	(0.00)	1	(2.85)	0	(0.00)	0	(0.00)	0	(0.00)
Skin toxicity	2	(5.71)	4	(11.42)	1	(2.85)	0	(0.00)	1	(2.85)	0	(0.00)	1	(2.85)	0	(0.00)
Sunburn	0	(0.00)	0	(0.00)	0	(0.00)	0	(0.00)	1	(2.85)	0	(0.00)	0	(0.00)	0	(0.00)
**Vascular disorders**	**1**	**(2.85)**	**1**	**(2.85)**	**1**	**(2.85)**	**0**	**(0.00)**	**0**	**(0.00)**	**1**	**(2.85)**	**1**	**(2.85)**	**0**	**(0.00)**
Hypertension	1	(2.85)	1	(2.85)	1	(2.85)	0	(0.00)	0	(0.00)	1	(2.85)	1	(2.85)	0	(0.00)

Supplementary Table 2. Drug related adverse events (AEs)

Footnote: Arm A: continuous schedule; Arm B: intermittent schedule. MedDRA version 16.0 and NCI CTCAE version 4.0 are used. No patients referred to death grade 5 events. Overall means the number of patients with an Adverse Events (AE) of the maximum grade. Multiple occurrences of the same AE in one individual counted only once per max grade.

		Arm A (N=35) n (%)	Arm B (N=35) n (%)	Total (N=70) n (%)
Dose Reduction	vemurafenib	15 (42.86)	14 (40.00)	29 (41.43)
	cobimetinib	17 (48.57)	12 (34.29)	29 (41.43)
Permanent Dose Interruption	vemurafenib	18 (51.43)	16 (45.71)	34 (48.57)
	cobimetinib	17 (48.57)	14 (40.00)	31 (44.29)
Temporary Dose Interruption	vemurafenib	23 (65.71)	21 (60.00)	44 (62.86)
	cobimetinib	22 (62.86)	19 (54.29)	41 (58.57)

Supplementary Table 3. Drug reductions and interruptions
Footnote: Arm A: continuous schedule; Arm B: intermittent schedule

	Arm A - Continuous-NEGATIVE	Arm A – Continuous-POSITIVE	Arm B - Intermittent-NEGATIVE	Arm B - Intermittent-POSITIVE
**Summary of events**				
No of patients	7	12	6	9
No of patients with event	3 (42.86%)	9 (75.00%)	2 (33.33%)	9 (100.00%)
No of censored patients	4 (57.14%)	3 (25.00%)	4 (66.67%)	0 (0.00%)
**Progression free survival**				
Median (95% CI)	NA (2.27, NA)	13.34 (4.64, NA)	NA (2.76, NA)	6.22 (0.33, 8.26)
25th-75th percentile	9.51 - NA	7.01 - NA	5.30 - NA	4.31 - 8.22
**Percent Survival (%, 95% CI)**				
0 Months	100.00 100.00, 100.00)	100.00 100.00, 100.00)	100.00 100.00, 100.00)	100.00 (100.00, 100.00)
12 Months	71.43 (25.82, 91.98)	58.33 (27.01, 80.09)	60.00 (12.57, 88.18)	11.11 (0.61, 38.77)
24 Months	57.14 (17.19, 83.71)	25.00 (6.01, 50.48)	60.00 (12.57, 88.18)	
36 Months	57.14 (17.19, 83.71)	25.00 (6.01, 50.48)	60.00 (12.57, 88.18)	

Supplementary Table 4. Progression free survival analysis according to BRAFV600 cfDNA and treatment arm

Footnote: Arm A: continuous schedule; Arm B: intermittent schedule; NEGATIVE: no BRAFV600 mutation in pretreatment cfDNA; POSITIVE: BRAFV600 mutation detectable in pretreatment cfDNA; cfDNA: tumoral cell free DNA

	Arm A – Continuous-NEGATIVE	Arm A – Continuous-POSITIVE	Arm B – Intermittent-NEGATIVE	Arm B – Intermittent-POSITIVE
**Summary of events**				
No of patients	7	12	6	9
No of patients with event	1 (14.29%)	8 (66.67%)	2 (33.33%)	8 (88.89%)
No of censored patients	6 (85.71%)	4 (33.33%)	4 (66.67%)	1 (11.11%)
**Overall survival**				
Median (95% CI)	NA (3.45, NA)	21.61 (5.39, NA)	NA (15.86, NA)	10.00 (0.33, 27.53)
25th-75th percentile	NA - NA	8.31 - NA	32.63 - NA	8.52 - 14.67
**Percent Survival (%, 95% CI)**				
0 Months	100.00 (100.00, 100.00)	100.00 (100.00, 100.00)	100.00 (100.00, 100.00)	100.00 (100.00, 100.00)
12 Months	85.71 (33.41, 97.86)	66.67 (33.70, 85.97)	100.00 (100.00, 100.00)	33.33 (7.83, 62.26)
24 Months	85.71 (33.41, 97.86)	29.17 (7.24, 56.09)	83.33 (27.31, 97.47)	22.22 (3.37, 51.31)
36 Months	85.71 (33.41, 97.86)	29.17 (7.24, 56.09)	66.67 (19.46, 90.44)	11.11 (0.61, 38.77)

Supplementary Table 5. Overall survival analysis according to BRAFV600 cfDNA and treatment arm

Footnote: Arm A: continuous schedule; Arm B: intermittent schedule; NEGATIVE: no BRAFV600 mutation in pretreatment cfDNA; POSITIVE: BRAFV600 mutation detectable in pretreatment cfDNA; cfDNA: tumoral cell free DNA

Variable			Stratified Kaplan-Meier model				Cox regression			
	**Stratum value**	**N**	**Events N(%)**	**Censored N(%)**	**Median (CI 95%)**	**Log-rank (p-value)**	**N**	**Contrast**	**Pr > ChiSq**	**Hazard Ratio (CI 95%)**
Basal BRAF	NEGATIVE	13	5 (38.46%)	8 (61.54%)	NA (2.76, NA)	0.0172	34	BRAF POSITIVE vs NEGATIVE	0.0234	3.217 (1.172, 8.831)
	POSITIVE	21	18 (85.71%)	3 (14.29%)	8.22 (5.20,13.62)	.	.		.	
LDH	LDH <= 2 UNL	29	18 (62.07%)	11 (37.93%)	13.78 (7.66, NA)	0.0019	34	LDH Elevated >2 UNL vs LDH <= 2 UNL	0.0046	4.546 (1.594, 12.968)
	LDH Elevated >2 UNL	5	5 (100.00%)	0 (0.00%)	4.64 (0.33,12.73)	.	.		.	
Age	<=60 YEARS	24	15 (62.50%)	9 (37.50%)	9.51 (6.35, NA)	0.3911	34	AGE >60 YEARS vs <=60 YEARS	0.3936	1.454 (0.615, 3.437)
	>60 YEARS	10	8 (80.00%)	2 (20.00%)	10.64 (0.33,20.03)	.	.		.	
Arm	A-Continuous	19	12 (63.16%)	7 (36.84%)	18.88 (7.66, NA)	0.0940	34	Arm: B-Intermittent vs A-Continuous	0.1002	2.004 (0.875, 4.591)
	B-Intermittent	15	11 (73.33%)	4 (26.67%)	7.50 (2.76,13.62)	.	.		.	
LDH	LDH <= 2.5 UNL	30	19 (63.33%)	11 (36.67%)	13.62 (7.66, NA)	<.0001	34	LDH Elevated >2.5 UNL vs LDH <= 2.5 UNL	0.0006	9.612 (2.657, 34.777)
	LDH Elevated >2.5 UNL	4	4 (100.00%)	0 (0.00%)	3.26 (0.33,6.22)	.	.		.	
LDH	LDH Elevated > UNL	15	14 (93.33%)	1 (6.67%)	7.50 (2.27,12.73)	0.0038	34	LDH Elevated > UNL vs LDH Within normal	0.0060	3.352 (1.414, 7.948)
	LDH Within normal limits	19	9 (47.37%)	10 (52.63%)	NA (7.66, NA)	.	.		.	

Supplementary Table 6. Univariate analysis of PFS – Sub-study population with basal BRAF cfDNA mITT population-

Footnote: Arm A: continuous schedule; Arm B: intermittent schedule; NEGATIVE: no BRAFV600 mutation in pretreatment cfDNA; POSITIVE: BRAFV600 mutation detectable in pretreatment cfDNA; cfDNA: tumoral cell free DNA; mITT: intention to treat

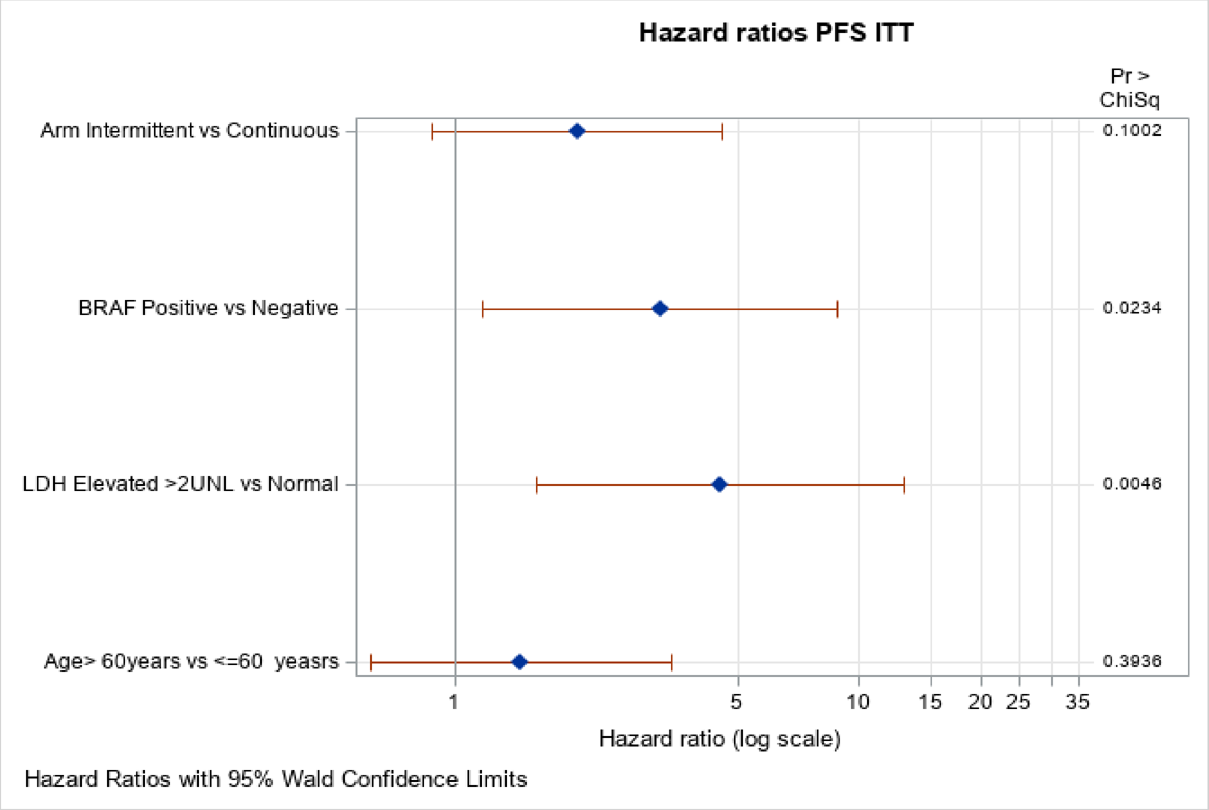
Hazard ratios PFS ITT
Pr > ChiSq
Arm Intermittent vs Continuous
0.1002
BRAF Positive vs Negative
0.0234
LDH Elevated >2UNL vs Normal
0.0046
Age> 60years vs <=60 yeasrs
0.3936
1
5
10
15
20
25
35
Hazard ratio (log scale)
Hazard Ratios with 95% Wald Confidence Limits

A

mITT		DF	Parameter Estimate	Standard Error	Chi-Square	Pr > ChiSq	HR	95% Hazard Ratio Confidence Limits	
**Arm**	**B-Intermittent**	1	0.77559	0.49633	2.4419	0.1181	2.172	0.821	5.745
**BRAF**	**POSITIVE**	1	1.61670	0.67627	5.7150	0.0168	5.036	1.338	18.957
**LDH**	**LDH Elevated >2 UNL**	1	1.40597	0.68278	4.2403	0.0395	4.079	1.070	15.552
**AGE**	**>60 YEARS**	1	1.07939	0.52956	4.1545	0.0415	2.943	1.042	8.309

Footnote: Arm B: intermittent schedule; POSITIVE: BRAFV600 mutation detectable in pretreatment cfDNA; cfDNA: tumoral cell free DNA; mITT: intention to treat; HR: hazard ratio

B

PP		DF	Parameter Estimate	Standard Error	Chi-Square	Pr > ChiSq	Hazard Ratio	95% Hazard Ratio Confidence Limits	
**Arm**	**B-Intermittent**	1	0.77559	0.49633	2.4419	0.1181	2.172	0.821	5.745
**BRAF**	**POSITIVE**	1	1.61670	0.67627	5.7150	0.0168	5.036	1.338	18.957
**LDH**	**LDH Elevated >2 UNL**	1	1.40597	0.68278	4.2403	0.0395	4.079	1.070	15.552
**AGE**	**>60 YEARS**	1	1.07939	0.52956	4.1545	0.0415	2.943	1.042	8.309

Supplementary Table 7. Multivariate analysis of PFS – Sub study population with basal BRAF cfDNA mITT (A) and PP population (B)-

Footnote: Arm B: intermittent schedule; POSITIVE: BRAFV600 mutation detectable in pretreatment cfDNA; cfDNA: tumoral cell free DNA; PP: per protocol population; HR: hazard ratio

Variable			Stratified Kaplan-Meier model				Cox regression			
	**Stratum value**	**N**	**Events N(%)**	**Censored N(%)**	**Median (CI 95%)**	**Log-rank (p-value)**	**N**	**Contrast**	**Pr > ChiSq**	**Hazard Ratio (CI 95%)**
Basal BRAF	NEGATIVE	13	3 (23.08%)	10 (76.92%)	NA (32.62, NA)	0.0024	34	BRAF POSITIVE vs NEGATIVE	0.0066	5.632 (1.617, 19.613)
	POSITIVE	21	16 (76.19%)	5 (23.81%)	14.68 (8.54,23.59)	.	.		.	
LDH	LDH <= 2 UNL	29	15 (51.72%)	14 (48.28%)	32.62 (14.68, NA)	0.0072	34	LDH Elevated >2 UNL vs LDH <= 2 UNL	0.0138	4.421 (1.354, 14.434)
	LDH Elevated >2 UNL	5	4 (80.00%)	1 (20.00%)	7.42 (0.36, NA)	.	.		.	
Age	<=60 YEARS	24	11 (45.83%)	13 (54.17%)	NA (10.02, NA)	0.0565	34	AGE >60 YEARS vs <=60 YEARS	0.0649	2.383 (0.948, 5.994)
	>60 YEARS	10	8 (80.00%)	2 (20.00%)	15.28 (0.36,23.00)	.	.		.	
Arm	A-Continuous	19	9 (47.37%)	10 (52.63%)	NA (9.76, NA)	0.3967	34	Arm: B-Intermittent vs A-Continuous	0.4007	1.472 (0.597, 3.631)
	B-Intermittent	15	10 (66.67%)	5 (33.33%)	15.87 (8.54, NA)	.	.		.	
LDH	LDH <= 2.5 UNL	30	15 (50.00%)	15 (50.00%)	32.62 (15.87, NA)	<.0001	34	LDH Elevated >2.5 UNL vs LDH <= 2.5 UNL	0.0006	9.710 (2.652, 35.560)
	LDH Elevated >2.5 UNL	4	4 (100.00%)	0 (0.00%)	6.42 (0.36,10.02)	.	.		.	
LDH	LDH Elevated > UNL	15	12 (80.00%)	3 (20.00%)	10.02 (3.48,21.62)	0.0018	34	LDH Elevated > UNL vs LDH Within normal	0.0038	4.097 (1.577, 10.641)
	LDH Within normal limits	19	7 (36.84%)	12 (63.16%)	NA (23.00, NA)	.	.		.	

Supplementary Table 8. Univariate analysis of OS – Sub study population with basal BRAF cfDNA mITT population-

Footnote: Arm A: continuous schedule; Arm B: intermittent schedule; NEGATIVE: no BRAFV600 mutation in pretreatment cfDNA; POSITIVE: BRAFV600 mutation detectable in pretreatment cfDNA; cfDNA: tumoral cell free DNA; mITT: intention to treat

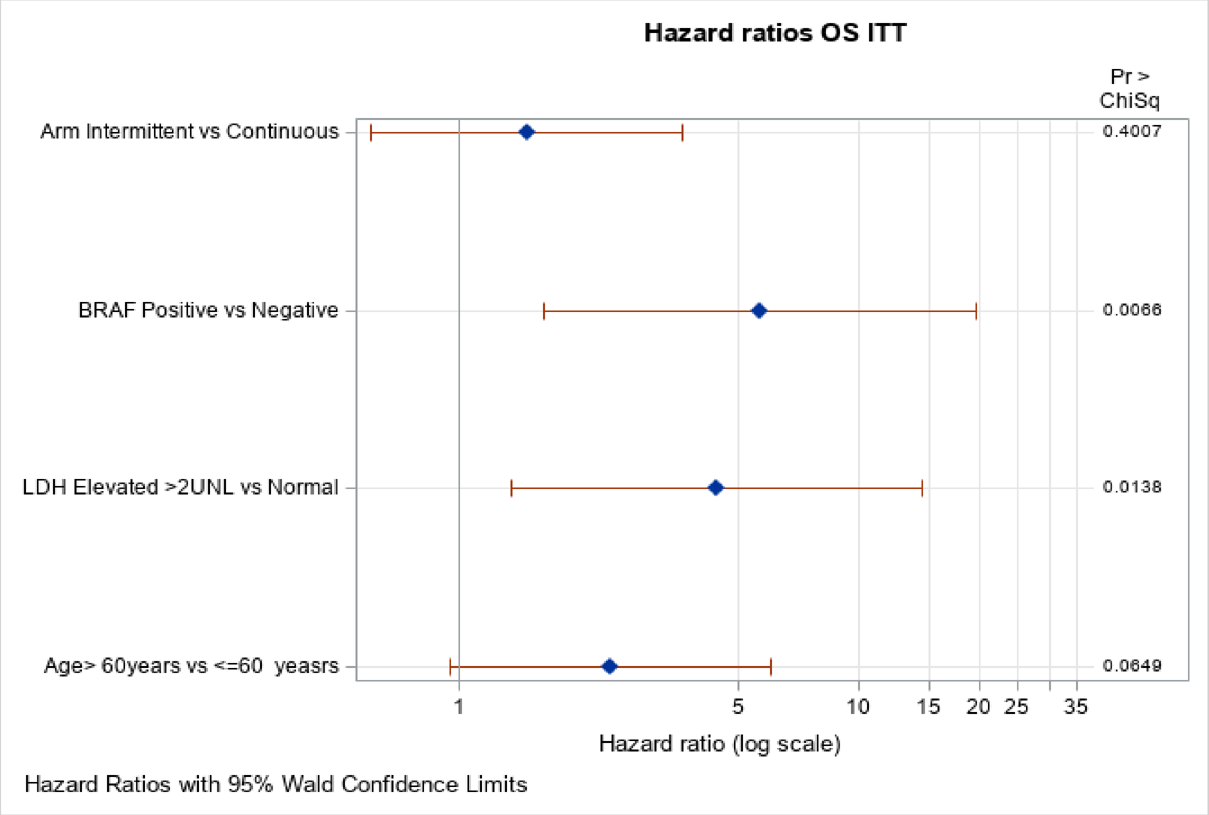
Hazard ratios OS ITT
Pr > ChiSq
Arm Intermittent vs Continuous
0.4007
BRAF Positive vs Negative
0.0066
LDH Elevated >2UNL vs Normal
0.0138
Age> 60years vs <=60 yeasrs
0.0649
1
5
10
15
20
25
35
Hazard ratio (log scale)
Hazard Ratios with 95% Wald Confidence Limits

A.

mITT		DF	Parameter Estimate	Standard Error	Chi-Square	Pr > ChiSq	Hazard Ratio	95% Hazard Ratio Confidence Limits	
Arm	B-Intermittent	1	0.77559	0.49633	2.4419	0.1181	2.172	0.821	5.745
BRAF	POSITIVE	1	1.61670	0.67627	5.7150	0.0168	5.036	1.338	18.957
LDH	LDH Elevated >2 UNL	1	1.40597	0.68278	4.2403	0.0395	4.079	1.070	15.552
AGE	>60 YEARS	1	1.07939	0.52956	4.1545	0.0415	2.943	1.042	8.309

Supplementary Table 9A. Multivariate analysis of OS – Sub study population with basal BRAF cfDNA mITT population

Footnote: Arm B: intermittent schedule; POSITIVE: BRAFV600 mutation detectable in pretreatment cfDNA; cfDNA: tumoral cell free DNA; mITT: intention to treat; HR: hazard ratio

B.

PP		DF	Parameter Estimate	Standard Error	Chi-Square	Pr > ChiSq	Hazard Ratio	95% Hazard Ratio Confidence Limits	
Arm	B-Intermittent	1	0.70347	0.60744	1.3412	0.2468	2.021	0.614	6.646
BRAF	POSITIVE	1	2.38610	1.06674	5.0033	0.0253	10.871	1.344	87.959
LDH	LDH Elevated >2 UNL	1	1.01308	0.88135	1.3213	0.2504	2.754	0.490	15.495
AGE	>60 YEARS	1	0.58595	0.68083	0.7407	0.3894	1.797	0.473	6.823

Supplementary Table 9B. Multivariate analysis of OS – Sub study population with basal BRAF cfDNA PP population

Footnote: Arm B: intermittent schedule; POSITIVE: BRAFV600 mutation detectable in pretreatment cfDNA; cfDNA: tumoral cell free DNA; PP: per protocol population; HR: hazard ratio

ARM A

ID Patient	Baseline BRAF V600	Progression BRAF V600	Treatment cycle	NGS Results	% Allelic fraction
GEM-0105	ND	ND	2	KIT Amplification; PDGFRA Amplification	
GEM-0207	V600E	V600E	17	BRAF: p.V600E; NRAS: pQ61R	BRAF (11%), NRAS (3%)
GEM-0601	V600E	V600E	15	BRAF: pV600E	BRAF (4,2%)
GEM-1801	V600E	ND	8	NRAS: pG12R	NRAS (1,9%)

ARM B

ID Patient	Baseline BRAF V600	Progression TAQMAN	Treatment cycle	NGS Results	% Allelic fraction
GEM-0203	V600E	V600E	2	BRAF:pV600E; TP53: p.F134L	BRAF (19%), TP53 (12%)
GEM-1005	V600E	V600E	6	BRAF: PV600E; NRAS: pQ61K	BRAF (18%), NRAS (22%)
GEM-1102	V600E	V600E	2	BRAF: p.V600E; PIK3CA: p.E545K; BRAF Amplification	BRAF (98%), PIK3CA (28%)
GEM-1802	V600E	V600E	4	BRAF: p.V600E; KRAS: p.G12V; NRAS: p.Q61K + p.Q61R	BRAF (18%), KRAS (5,7%), NRAS p.Q61K (7,25%), p.Q61R (4,9%)

Supplementary Table 10. Results of NGS analysis at Progression by treatment arm

Footnote: Arm A: continuous schedule; Arm B: intermittent schedule; Basal TAQMAN: results by Taqman analysis of BRAFV600 mutation in pretreatment cfDNA; V600E: BRAFV600 mutation detectable in cfDNA; ND: no detected; Treatment cycle: Number of cycle when progression sample was analyzed; NGS: Next generation analysis in blood samples taken at disease progression

EXTENDED DATA FIGURES

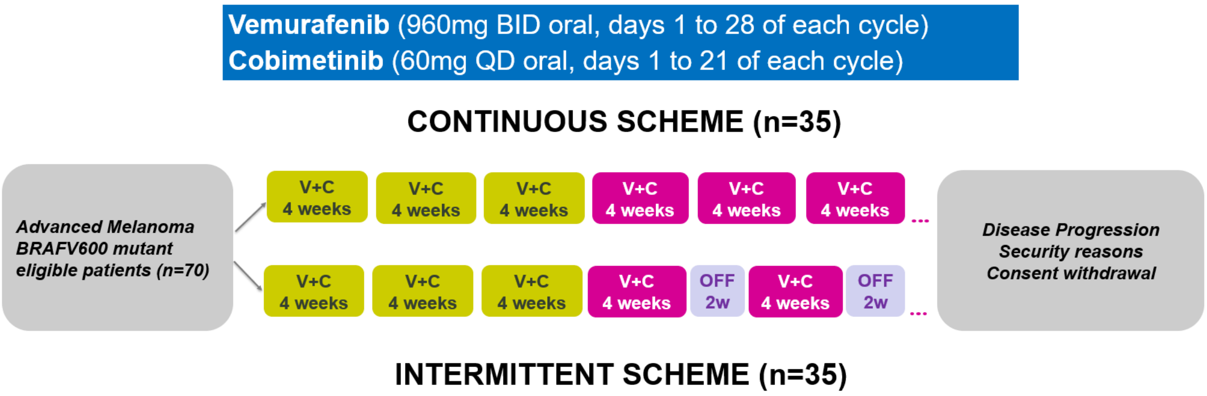

Supplementary Figure 1. Treatment schedule

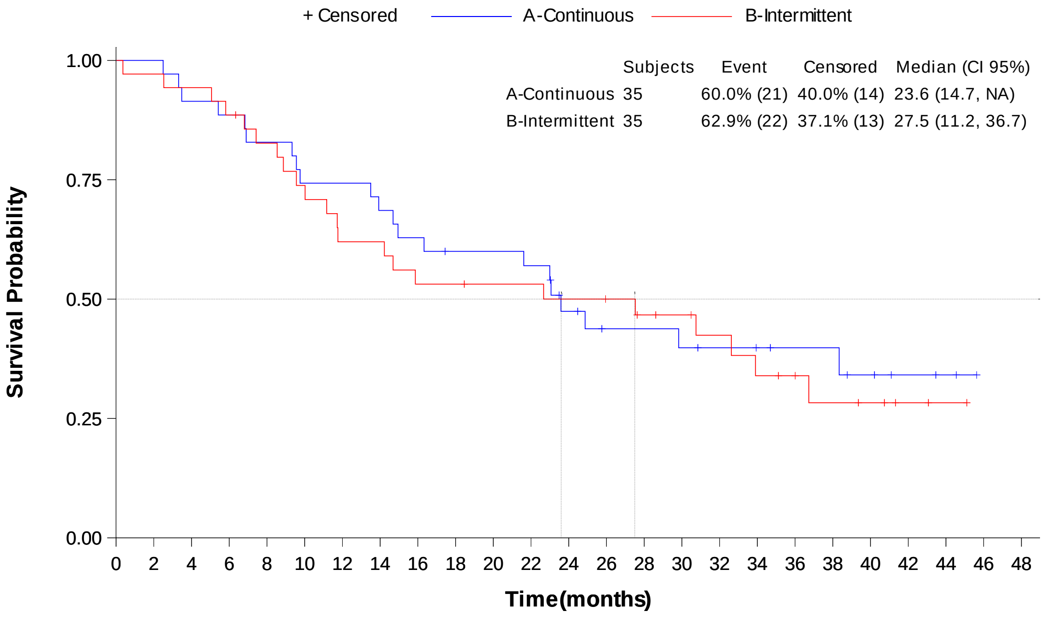

Patients at Risk

A-Continuous	35	35	32	31	29	26	26	24	22	20	20	19	14	11	11	10	9	8	7	7	5	3	2	0	0
B-Intermittent	35	34	33	31	28	25	21	21	18	18	17	17	16	15	13	12	10	8	7	5	4	2	1	0	0

**Overall Survival (%, 95% CI)**		
12 Months	74.29 (56.40, 85.70)	62.00 (43.68, 75.89)
24 Months	47.33 (29.93, 62.88)	50.02 (32.40, 65.32)
36 Months	39.95 (23.13, 56.27)	33.95 (17.61, 51.07)

Supplementary Figure 2. Overall survival of BRAFV600 mutant melanoma patients by treatment arm (A continuous schedule, B intermittent schedule). Median overall survival (OS) was 23.59 months (95% CI 14.67- NA) in Group A- Continuous versus 27.53 months (95% CI 11.15 - NA) in Group B- Intermittent, and was statistically non-significant (p= 0.7293). *Overall survival was estimated by means of the Kaplan–Meier method and the nonparametric two-side log-rank test was applied for comparisons of groups.*

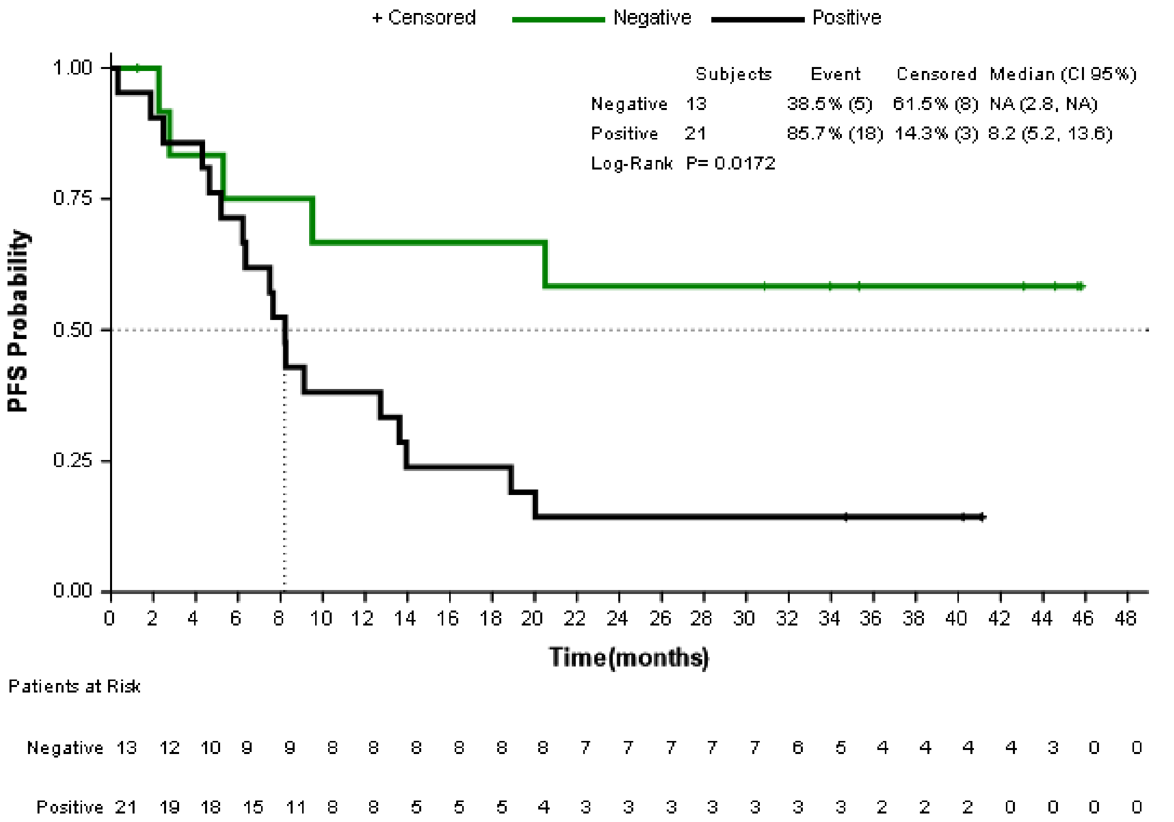

Supplementary Figure 3. A. Progression free survival according to the presence of BRAFV600 mutation in pretreatment cfDNA. There were significant differences in terms of median progression free survival according to BRAF detection in basal cfDNA with a median PFS not reached (95% CI 2.76, NR) in patients without BRAF detection (preBRAF-) versus 14.67 months (95% CI 8.52, 23.59) in preBRAF+, p=0.0518**.** *Progression free survival was estimated by means of the Kaplan–Meier method and the nonparametric two-side log-rank test was applied for comparisons of groups.*

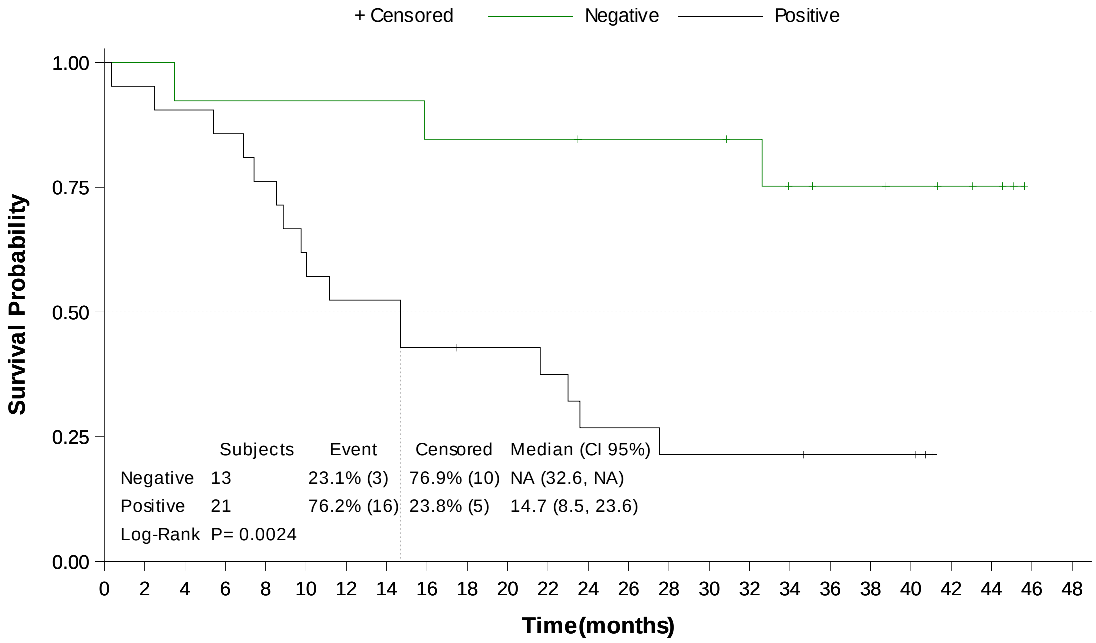

Patients at Risk

Negative	13	13	12	12	12	12	12	12	11	11	11	11	10	10	10	10	9	7	6	6	5	4	3	0	0
Positive	21	20	19	18	16	13	11	11	9	8	8	7	5	5	4	4	4	4	3	3	3	0	0	0	0

Supplementary Figure 3. B. Overall survival according to the presence of BRAFV600 mutation in pretreatment cfDNA. There were significant differences in terms of median survival according to BRAF detection in basal cfDNA with a median OS not reached (95% CI 32.63, NR) in patients preBRAF- versus 8.26 months in preBRAF+ (95% CI 5.20, 18.88), p=0.0024**.** *Overall survival was estimated by means of the Kaplan–Meier method and the nonparametric two-side log-rank test was applied for comparisons of groups.*

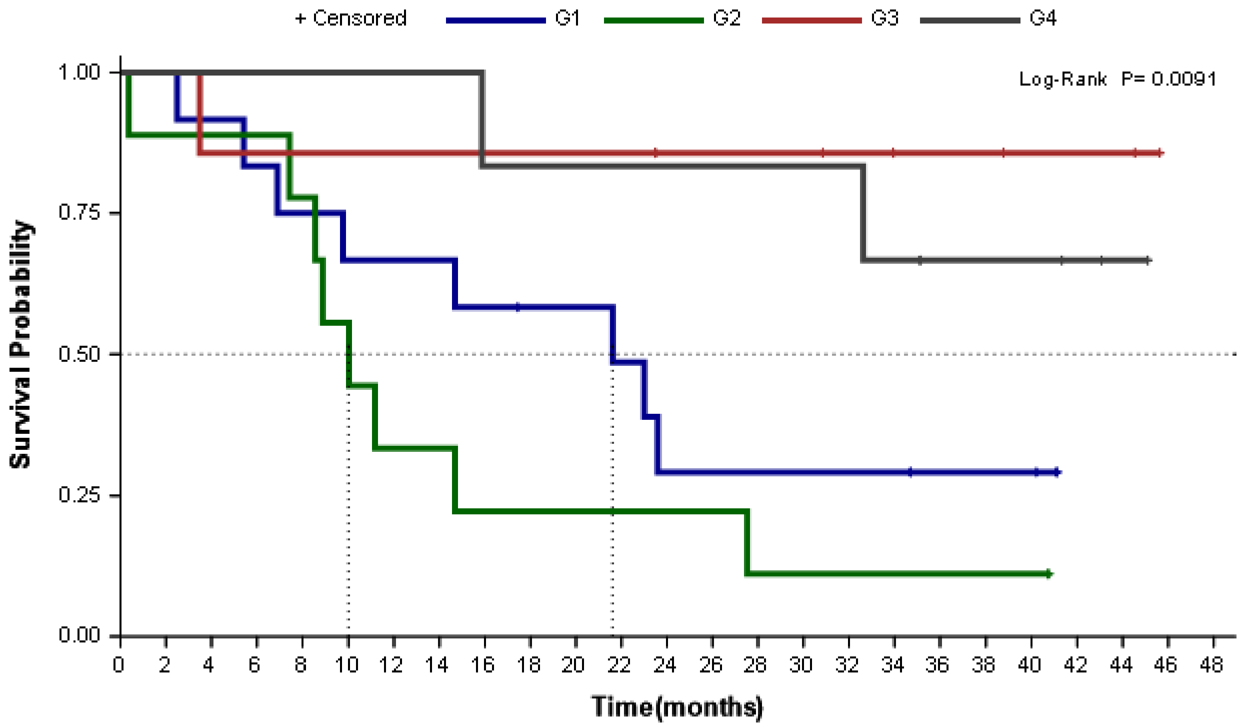

Patients at Risk

G1	12	12	11	10	9	8	8	8	7	6	6	5	3	3	3	3	3	3	2	2	2	0	0	0	0
G2	9	8	8	8	7	5	3	3	2	2	2	2	2	2	1	1	1	1	1	1	1	0	0	0	0
G3	7	7	6	6	6	6	6	6	6	6	6	6	5	5	5	5	4	3	3	3	2	2	2	0	0
G4	6	6	6	6	6	6	6	6	5	5	5	5	5	5	5	5	5	4	3	3	3	2	1	0	0

G1: Arm A, BRAF+
G2: Arm B, BRAF+
G3: Arm A, BRAF-
G4: Arm B, BRAF-

Supplementary Figure 4. Overall survival according to the presence of BRAFV600 mutation in pretreatment cfDNA and treatment arm. There were significant differences in terms of median survival according to BRAF detection in basal cfDNA in each treatment arm. In the continuous arm, median OS was 21.6 months (95% CI 5.4,NR) in preBRAF+, and NR for preBRAF- (95% CI 3.5,NR). For the intermittent arm, median OS was 10 months (95% CI 0.4-27.5) for preBRAF+, and NR for preBRAF- (95%CI 15.9, NR) (p=0.009). *Overall survival was estimated by means of the Kaplan–Meier method and the nonparametric two-side log-rank test was applied for comparisons of groups.*

Footnote:
G1: Continuous arm and basal BRAF Positive in cfDNA, G2: Intermittent arm and basal BRAF Positive in cfDNA, G3: Continuous arm and basal BRAF Negative in cfDNA, G4: Intermittent arm and basal BRAF Negative in cfDNA

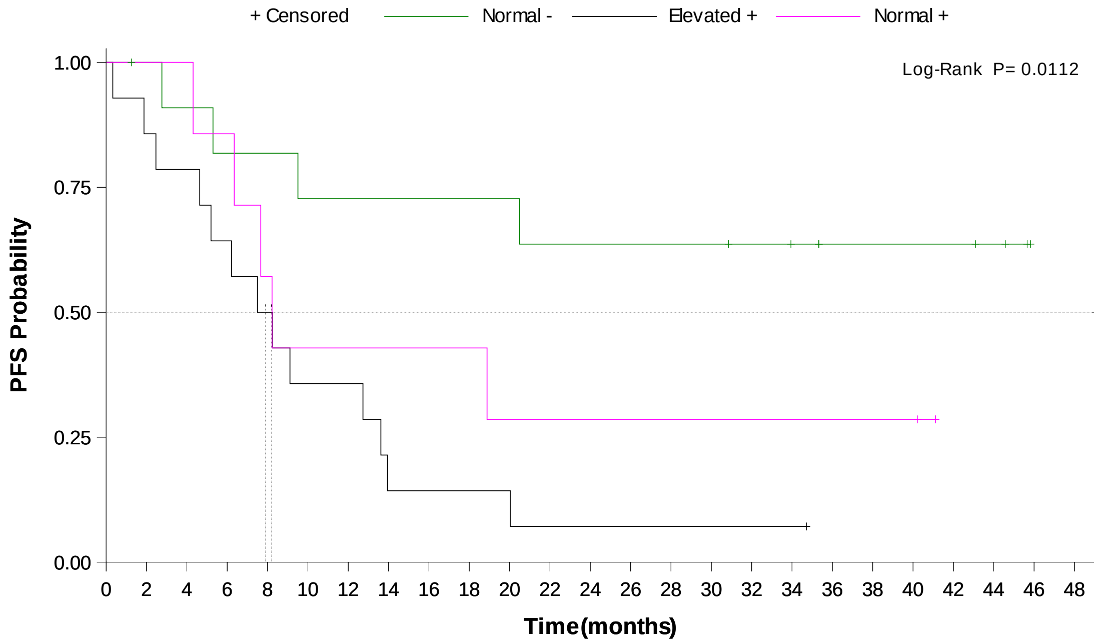

Patients at Risk

	0	2	4	6	8	10	12	14	16	18	20	22	24	26	28	30	32	34	36	38	40	42	44	46	48
Normal -	12	11	10	9	9	8	8	8	8	8	8	7	7	7	7	7	6	5	4	4	4	4	3	0	0
Elevated +	14	12	11	9	7	5	5	2	2	2	2	1	1	1	1	1	1	1	0	0	0	0	0	0	0
Normal +	7	7	7	6	4	3	3	3	3	3	2	2	2	2	2	2	2	2	2	2	2	0	0	0	0

Kaplan-Meier model- Summary results

Strata	Subjects	Event	% Events	Censored	% Censored	Median	CI 95% LL	CI 95% UL
Elevated +	14	13	92.9	1	7.1	7.9	2.5	13.6
Normal +	7	5	71.4	2	28.6	8.2	4.3	.
Normal -	12	4	33.3	8	66.7	.	5.3	.

Log-rank test

Test	Chi-Square	DF	Pr > Chi-Square
Log-Rank	8.9897	2	0.0112

Supplementary Figure 5. Progression free survival according to pretreatment LDH levels and the presence of BRAFV600 mutation in pretreatment cfDNA. There were significant differences in terms of median progression free survival according to BRAF detection in basal cfDNA in patients with normal LDH levels. There was only one patient with LDH Elevated and basal BRAF Negative (id=0105) . For this reason, the group Elevated - does not appear in the survival analysis. Patients with high LDH levels had a median PFS of 7.9 months (95% CI 2.5,13.6), while patients with normal LDH levels and BRAF detection in pretreatment cfDNA had a median PFS of 8.2 months (95% CI 4.3,NR) and patients with normal LDH levels and no BRAF detection in cfDNA had NR months (95% CI 5.3,NR) (p=0.0112). *Progression free survival was*

estimated by means of the Kaplan–Meier method and the nonparametric two-side log-rank test was applied for comparisons of groups.

Footnote: Normal -: Normal LDH levels and BRAF negative in cfDNA pretreatment; Normal +: Normal LDH levels and BRAF positive in cfDNA pretreatment; Elevated +: High LDH levels and BRAF positive in cfDNA pretreatment

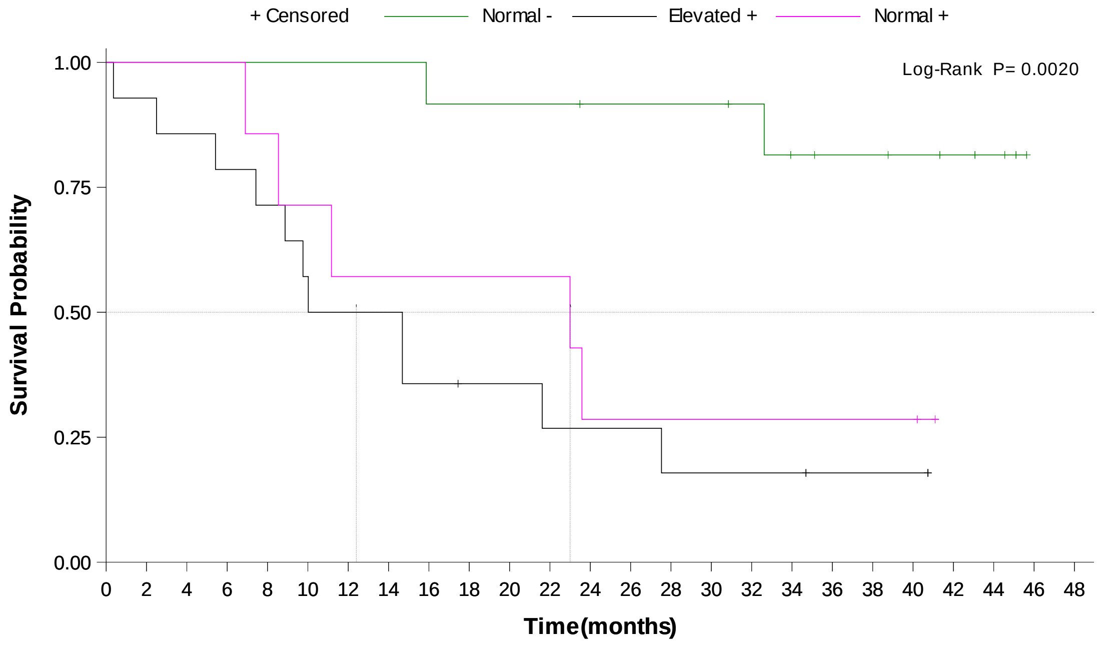

Patients at Risk

Normal -	12	12	12	12	12	12	12	12	11	11	11	11	10	10	10	10	9	7	6	6	5	4	3	0	0
Elevated +	14	13	12	11	10	8	7	7	5	4	4	3	3	3	2	2	2	2	1	1	1	0	0	0	0
Normal +	7	7	7	7	6	5	4	4	4	4	4	4	2	2	2	2	2	2	2	2	2	0	0	0	0

Kaplan-Meier model- Summary results

Strata	Subjects	Event	% Events	Censored	% Censored	Median	CI 95% LL	CI 95% UL
Elevated +	14	11	78.6	3	21.4	12.4	5.4	27.5
Normal +	7	5	71.4	2	28.6	23.0	6.9	.
Normal -	12	2	16.7	10	83.3	.	32.6	.

Log-rank test

Test	Chi-Square	DF	Pr > Chi-Square
Log-Rank	12.4623	2	0.0020

Supplementary Figure 6. Overall survival according to pretreatment LDH levels and the presence of BRAFV600 mutation in pretreatment cfDNA. There were significant differences in terms of median overall survival according to BRAF detection on basal cfDNA in patients with normal LDH levels. There was only one patient withLDH elevated and basal BRAF Negative (id=0105). For this reason, the group Elevated - does not appear in the survival analysis. Patients with high LDH levels had a median

OS of 12.4 months (95% CI 5.4,27.5), while for patients with normal LDH levels and BRAF detection in pretreatment cfDNA, median OS was 23 months (95% CI 6.9,NR), and for patients with normal LDH levels and no BRAF detection in cfDNA, it was NR months (95% CI 32.6,NR) (p=0.0020). *Overall survival was estimated by means of the Kaplan–Meier method and the nonparametric two-side log-rank test was applied for comparisons of groups.*

Footnote: Normal -: Normal LDH levels and BRAF negative in cfDNA pretreatment; Normal +: Normal LDH levels and BRAF positive in cfDNA pretreatment; Elevated +: High LDH levels and BRAF positive in cfDNA pretreatment

BRAF cfDNA (% mutated allele) and best response- pt=205 TRT A

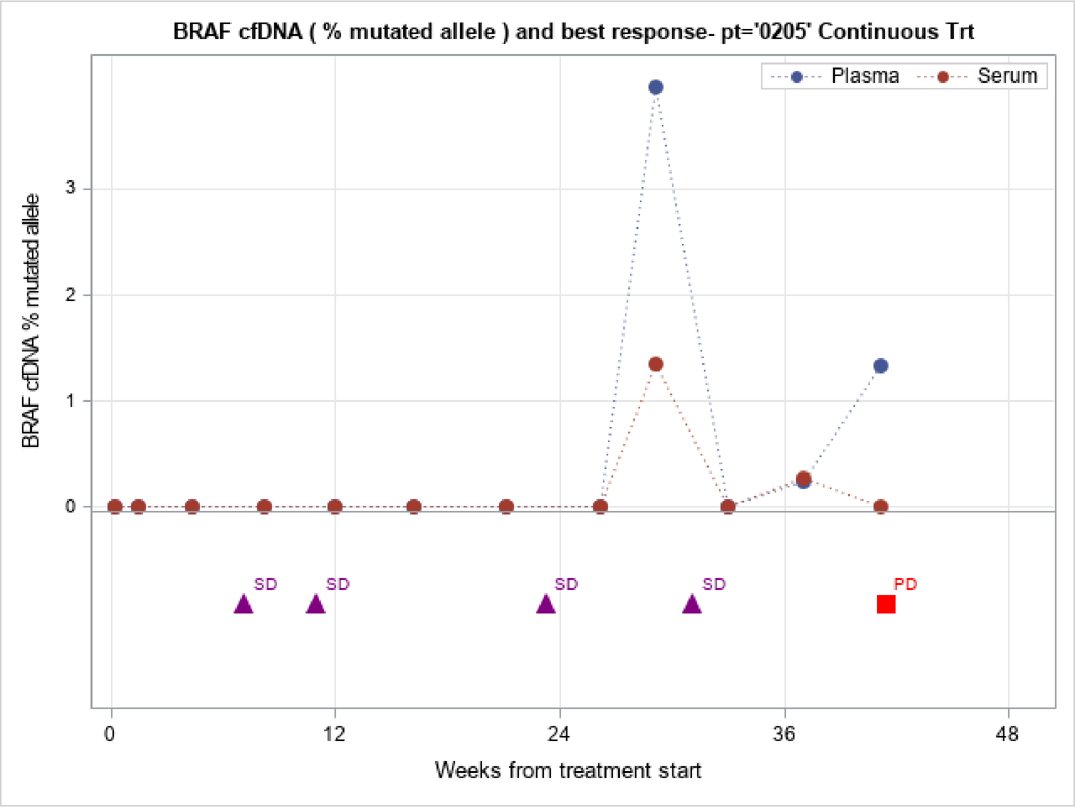

BRAF cfDNA (pg/□L mutated allele) and best response- pt=205 TRT A

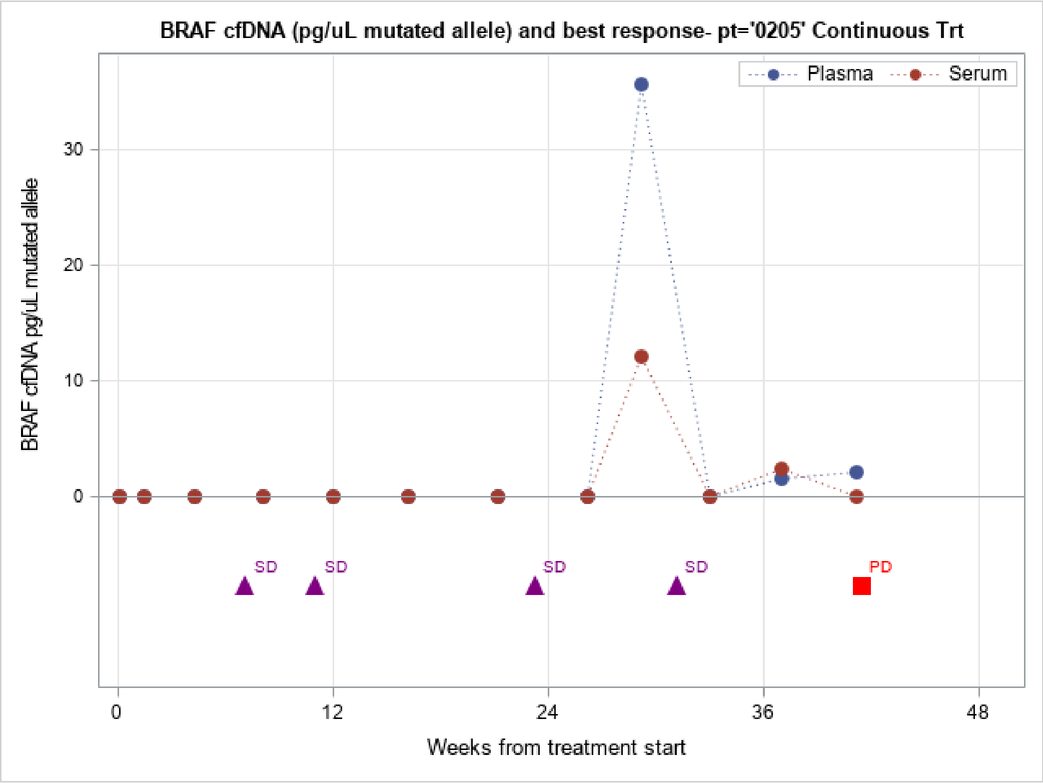

BRAF cfDNA (% mutated allele) and best response- pt=206 TRT A

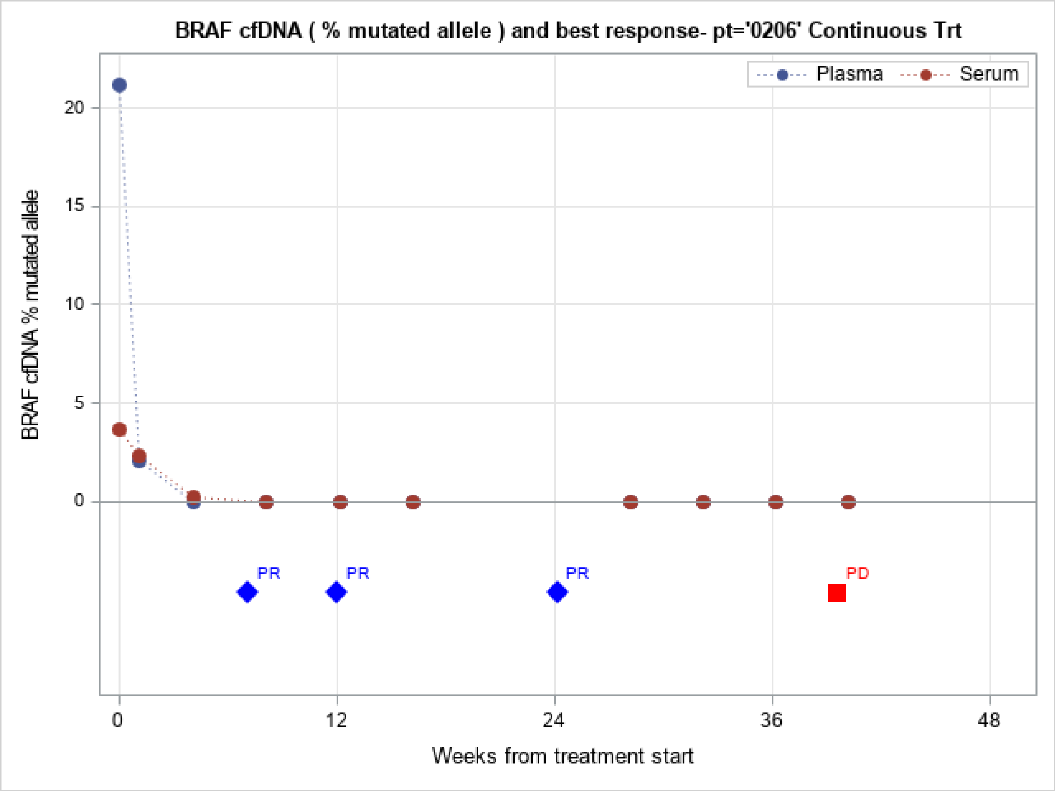

BRAF cfDNA (pg/□L mutated allele) and best response- pt=206 TRT A

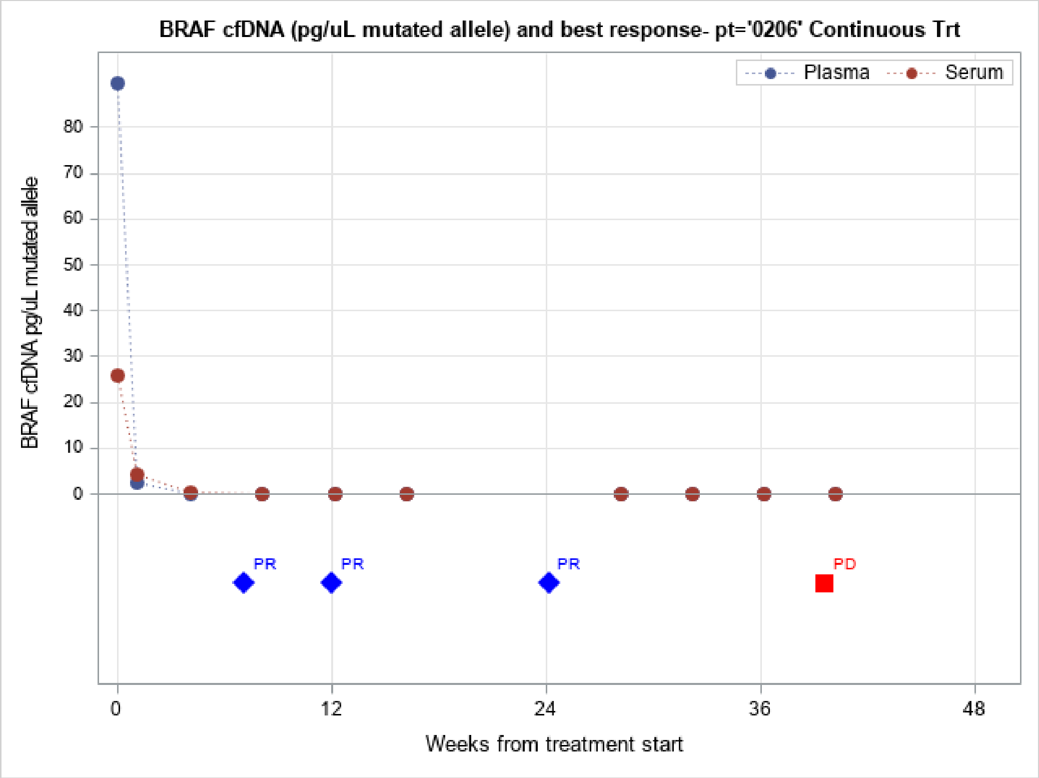

BRAF cfDNA (% mutated allele) and best response- pt=207 TRT A

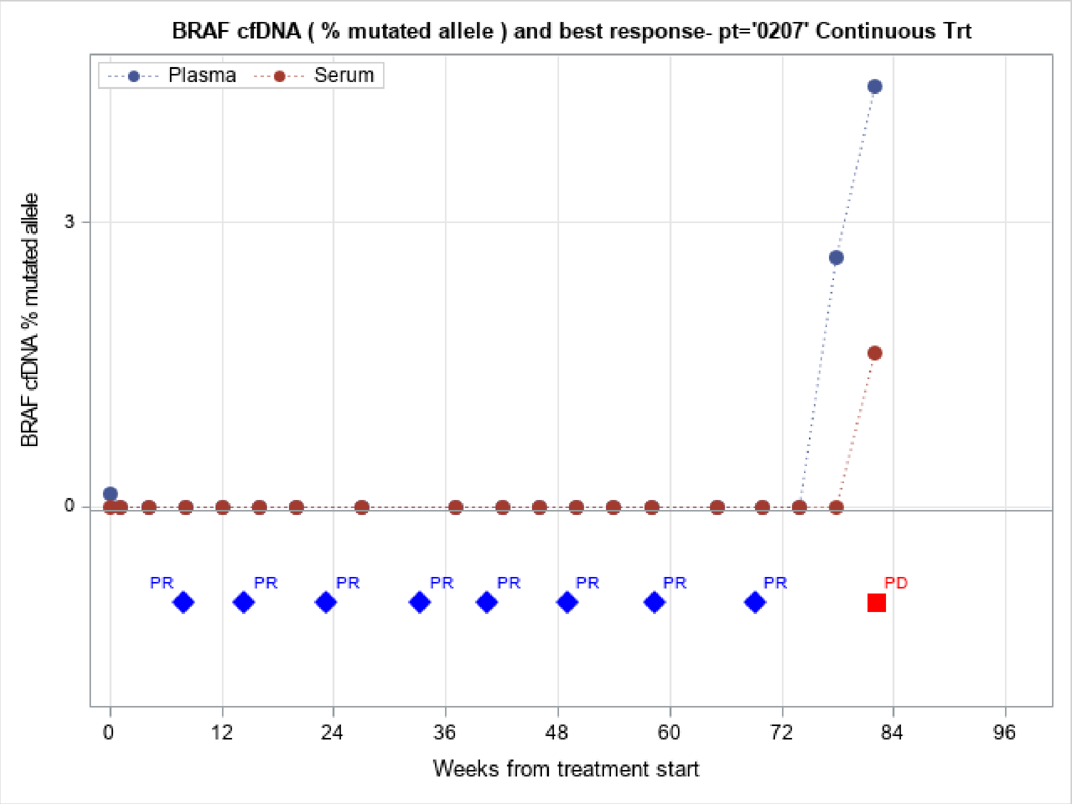

BRAF cfDNA (pg/□L mutated allele) and best response- pt=207 TRT A

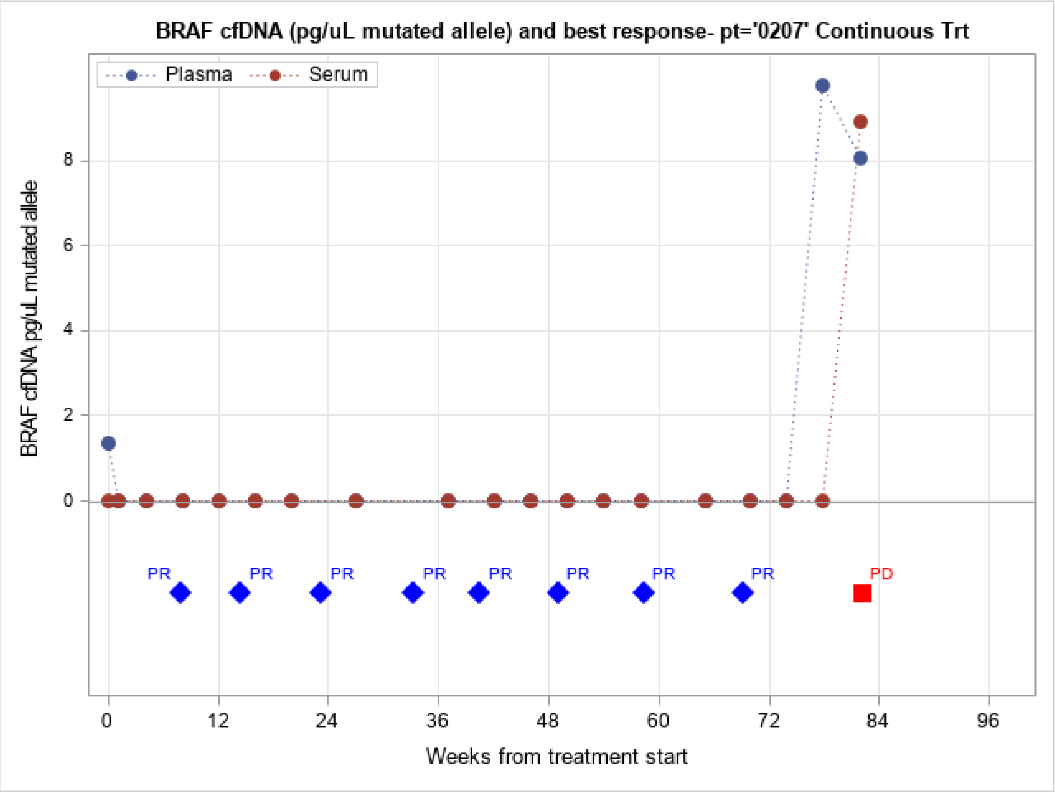

BRAF cfDNA (% mutated allele) and best response- pt=401 TRT A

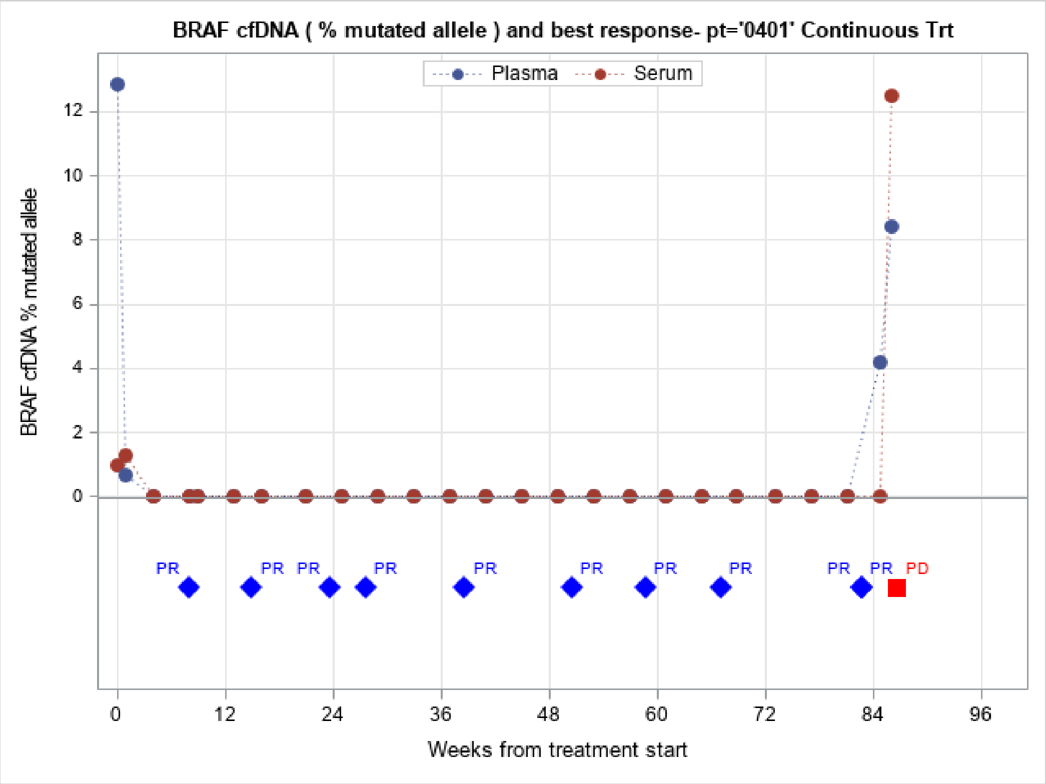

BRAF cfDNA (pg/□L mutated allele) and best response- pt=401 TRT A

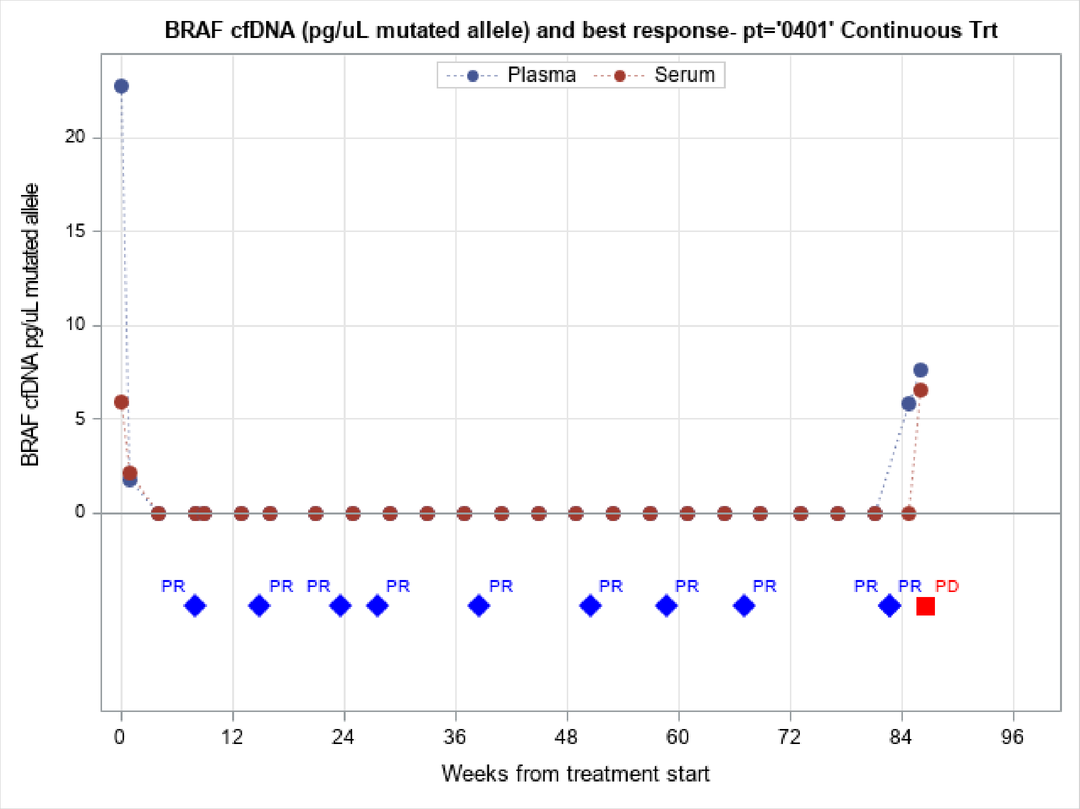

BRAF cfDNA (% mutated allele) and best response- pt=601 TRT A

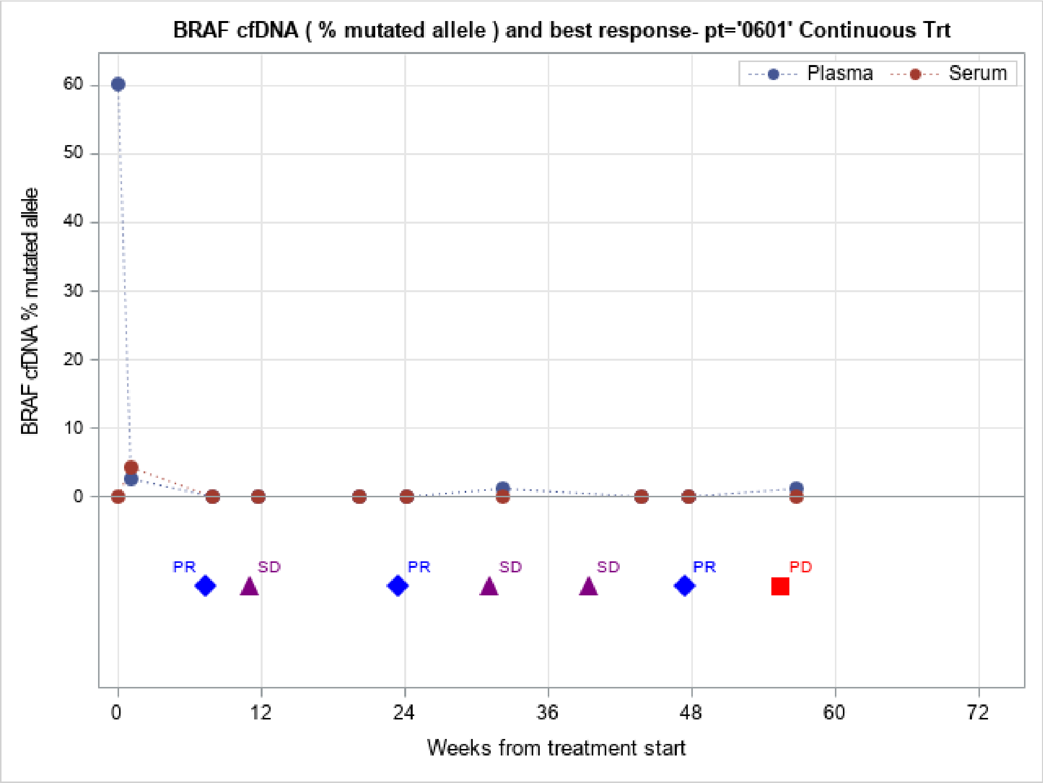

BRAF cfDNA (pg/□L mutated allele) and best response- pt=601 TRT A

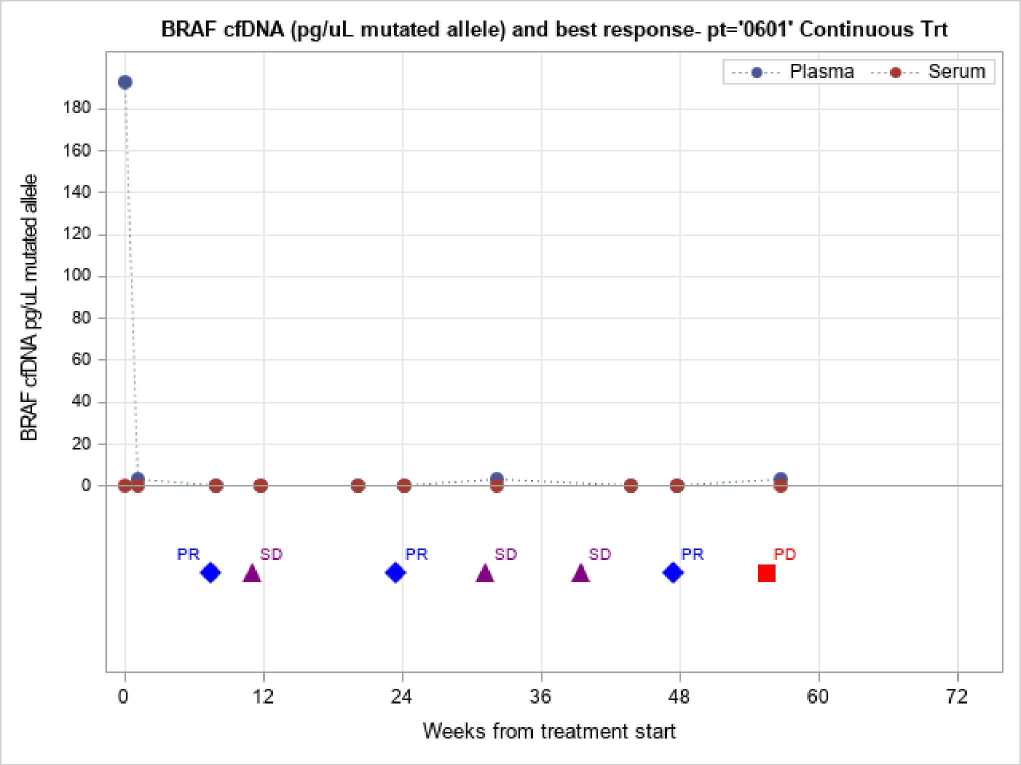

BRAF cfDNA (% mutated allele) and best response- pt=1104 TRT A

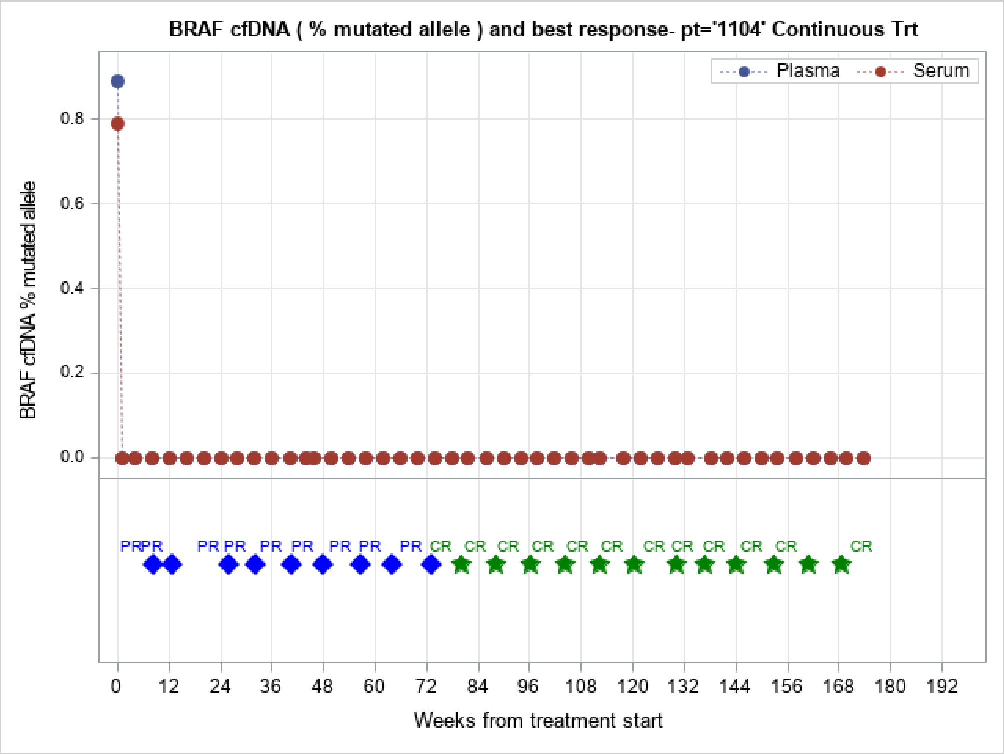

BRAF cfDNA (pg/□L mutated allele) and best response- pt=1104 TRT A

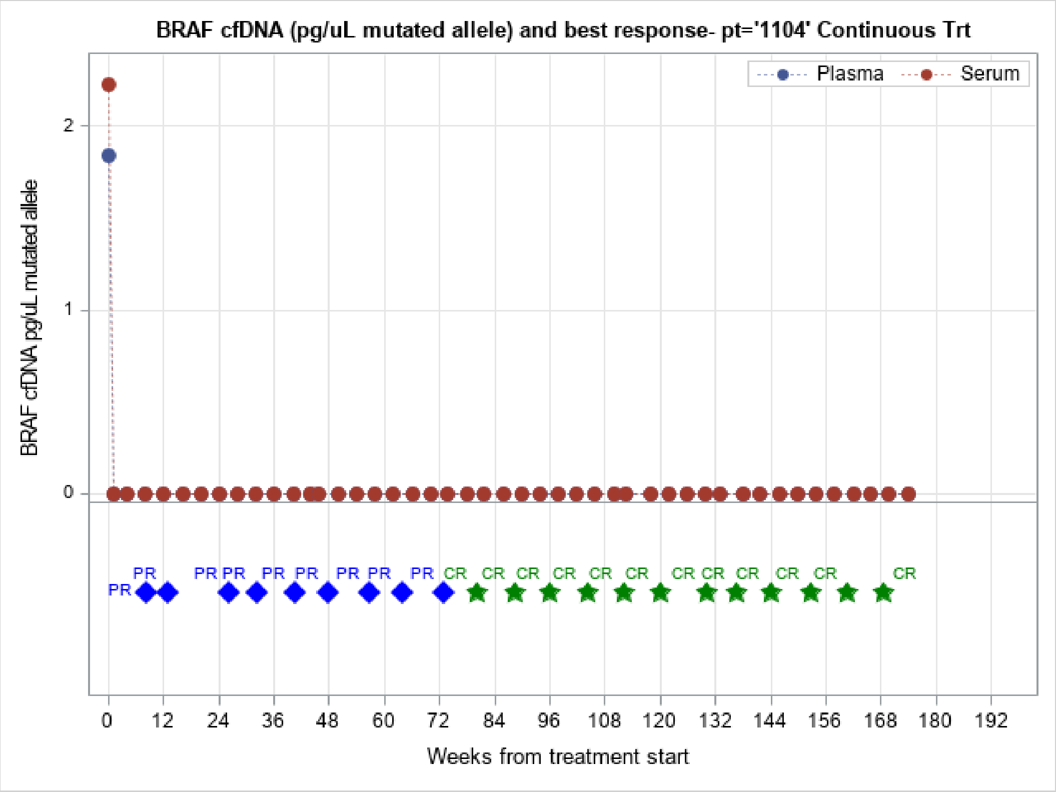

BRAF cfDNA (% mutated allele) and best response- pt=1106 TRT A

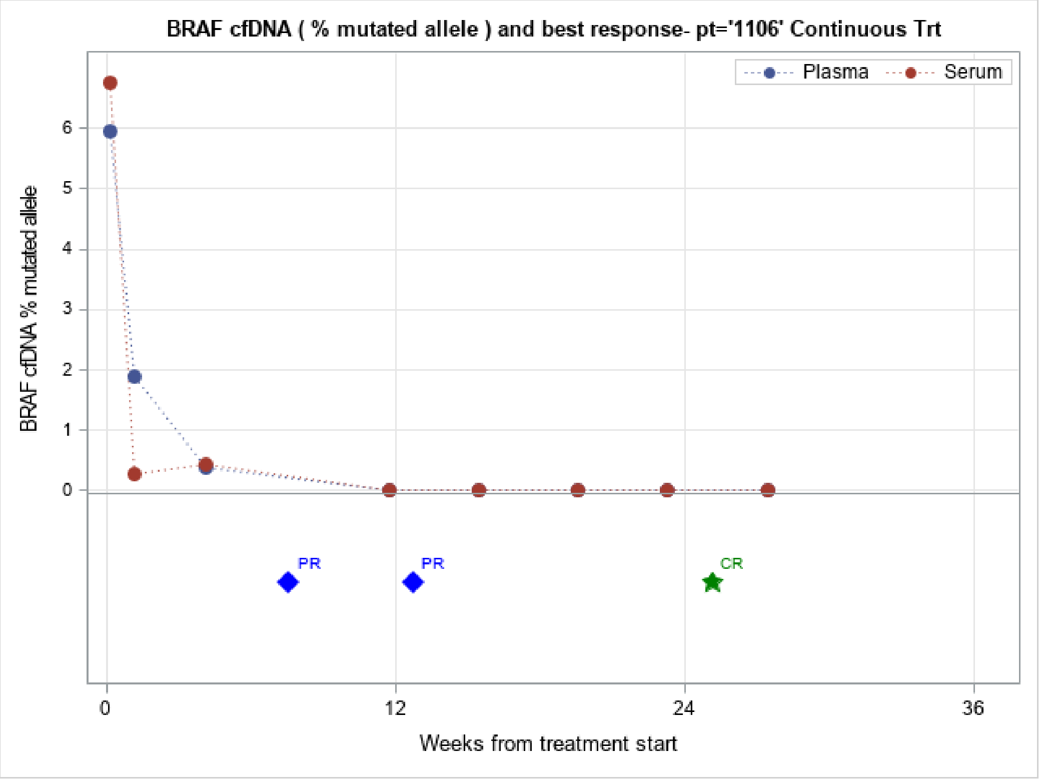

BRAF cfDNA (pg/□L mutated allele) and best response- pt=1106 TRT A

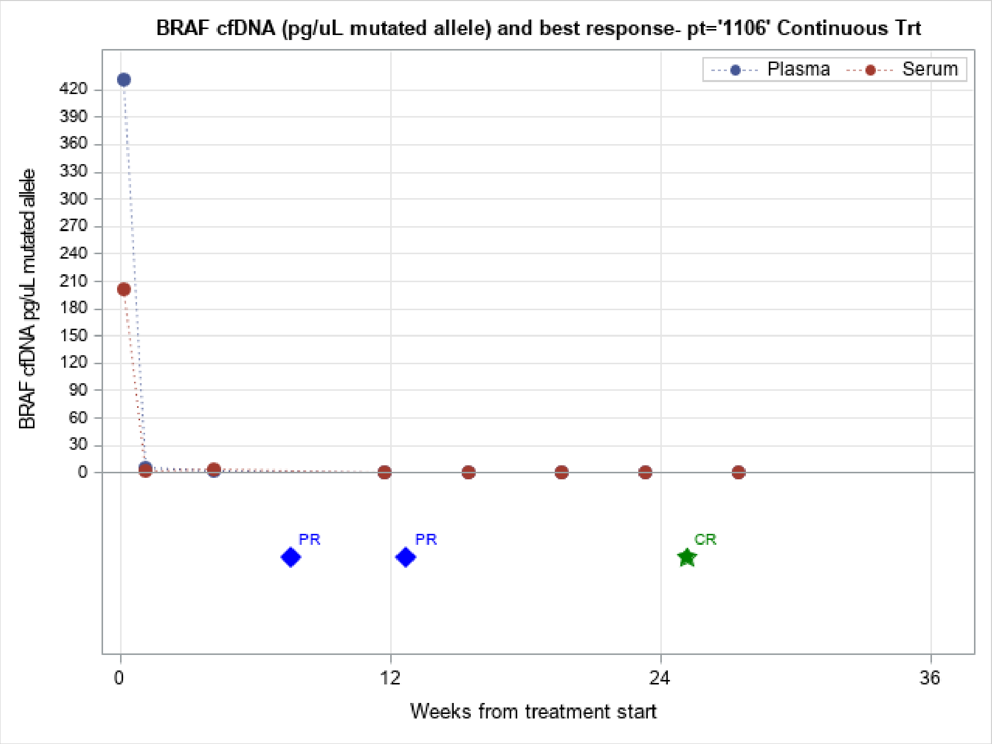

BRAF cfDNA (% mutated allele) and best response- pt=203 TRT B

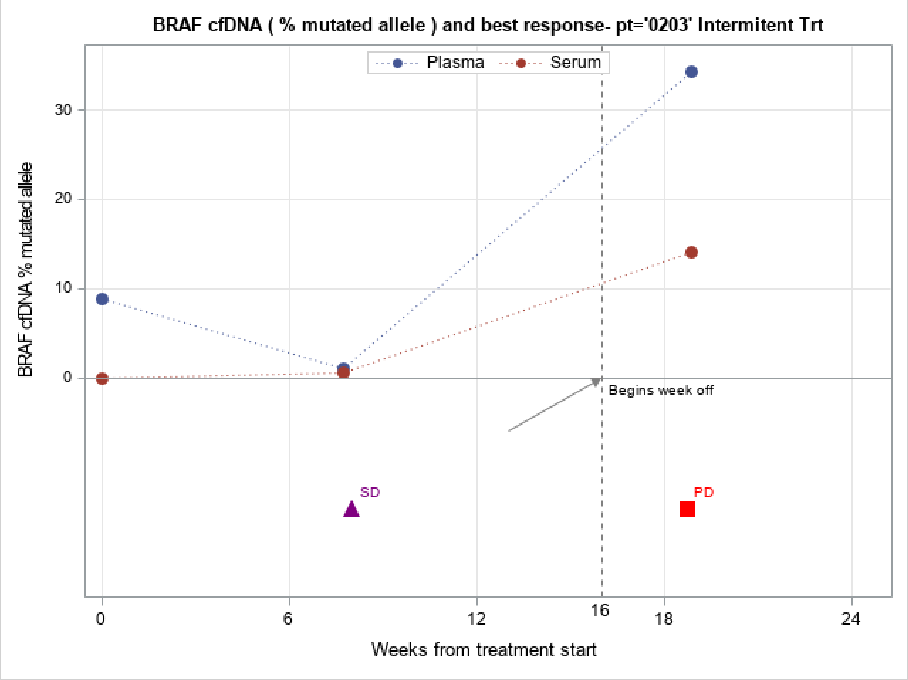

BRAF cfDNA (pg/□L mutated allele) and best response- pt=203 TRT B

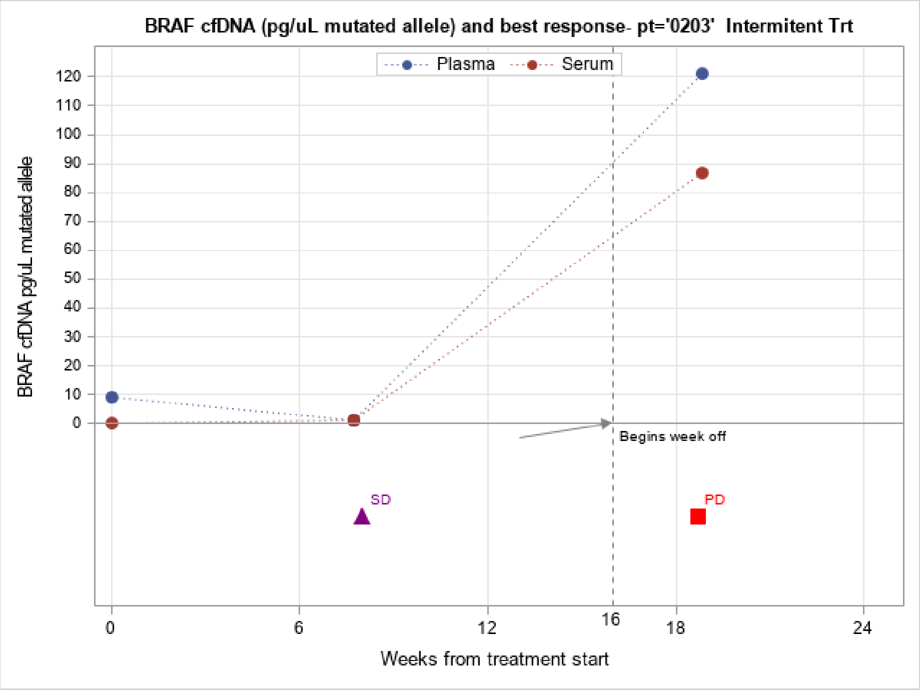

BRAF cfDNA (% mutated allele) and best response- pt=406 TRT B

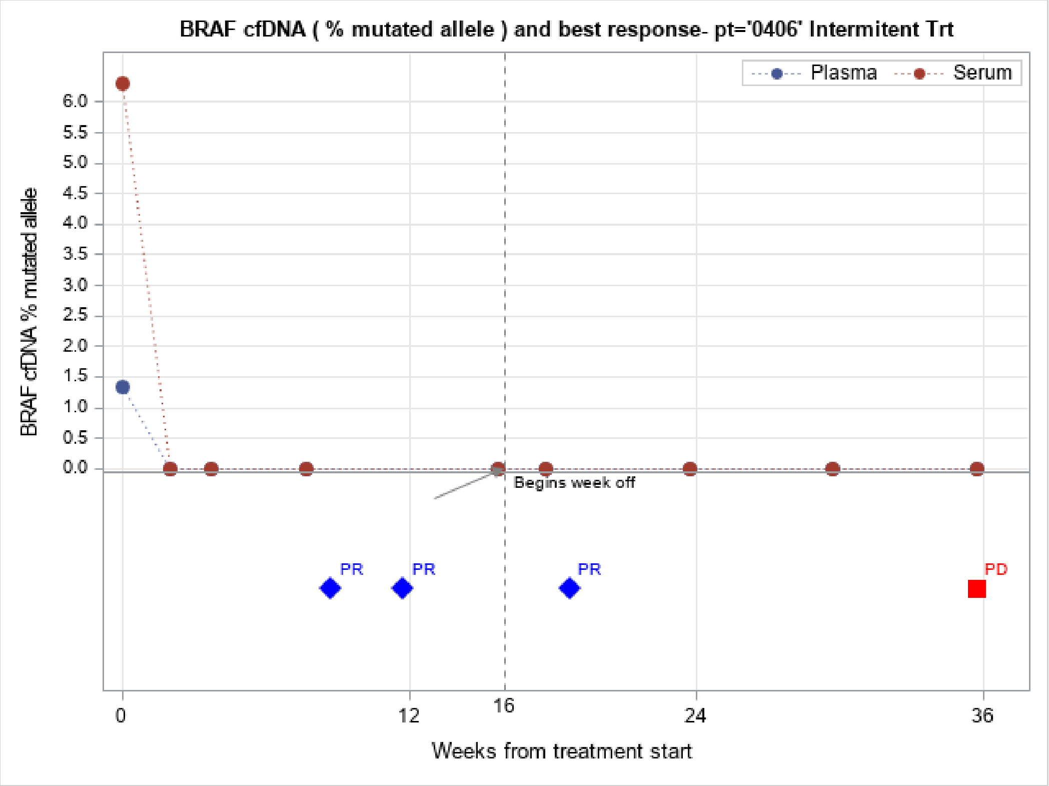

BRAF cfDNA (pg/□L mutated allele) and best response- pt=406 TRT B

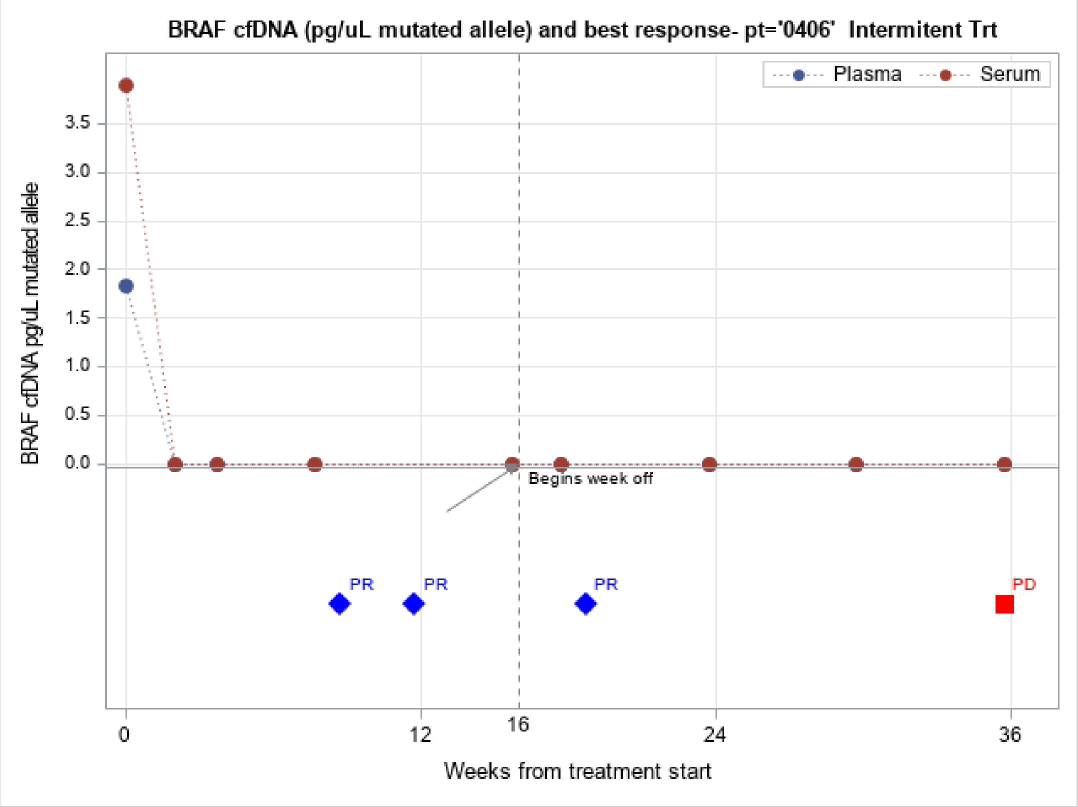

BRAF cfDNA (% mutated allele) and best response- pt=701 TRT B

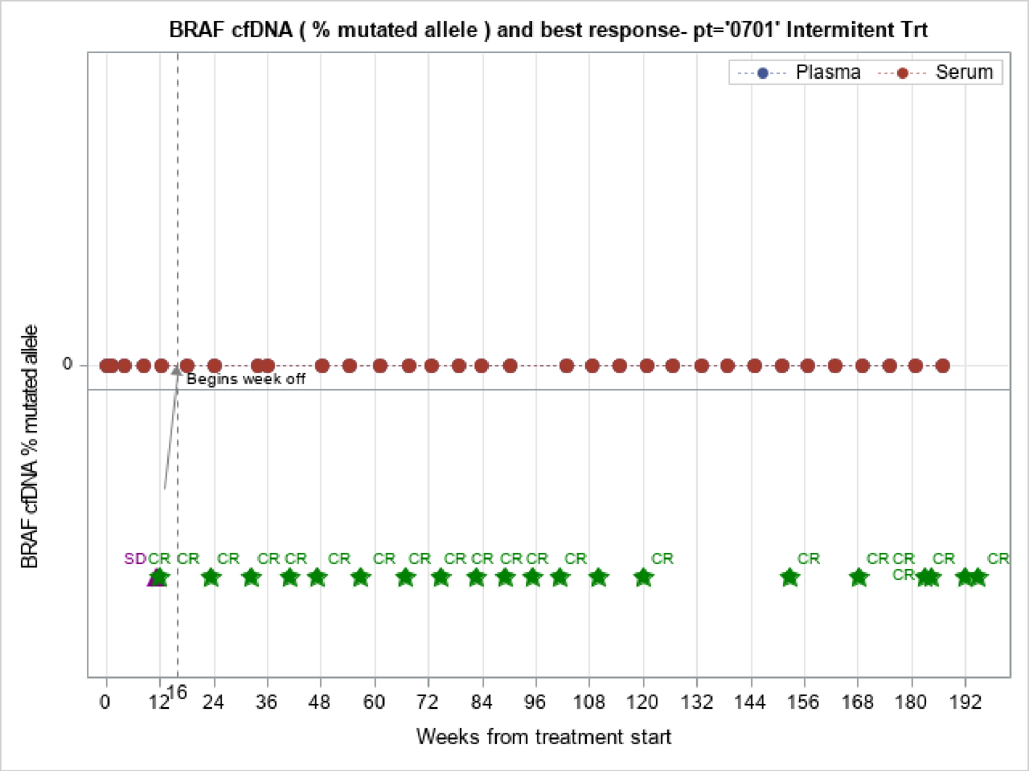

BRAF cfDNA (pg/□L mutated allele) and best response- pt=701 TRT B

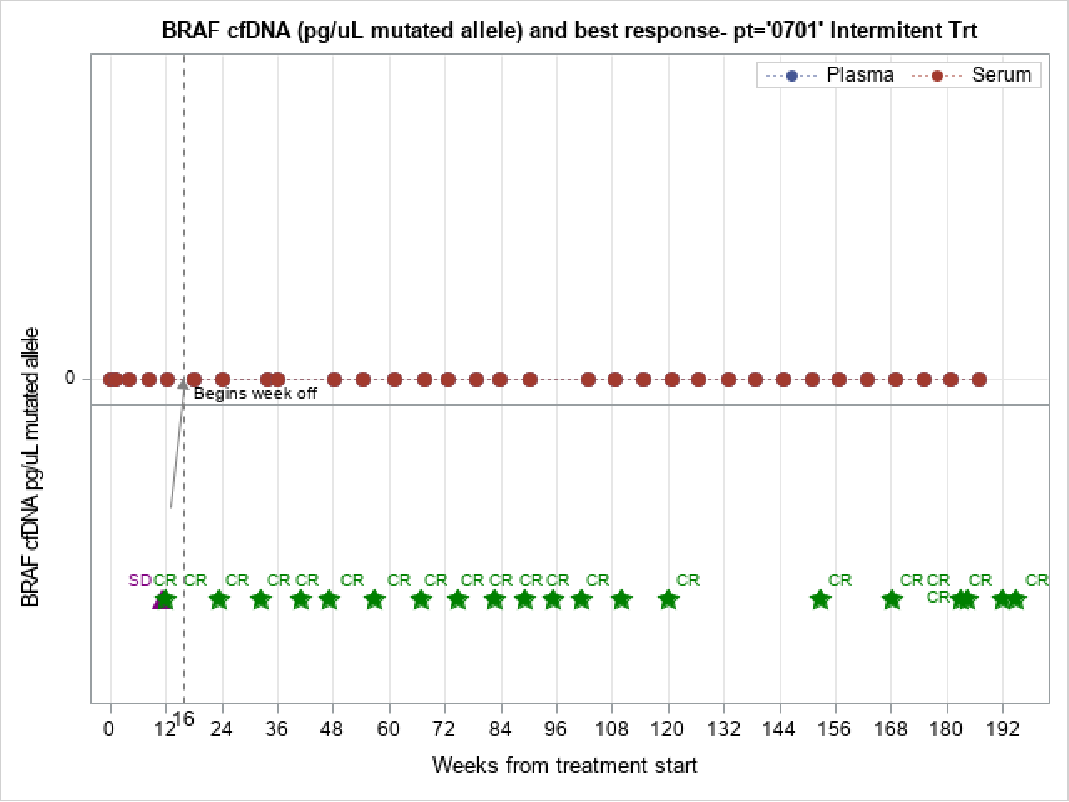

BRAF cfDNA (% mutated allele) and best response- pt=703 TRT B

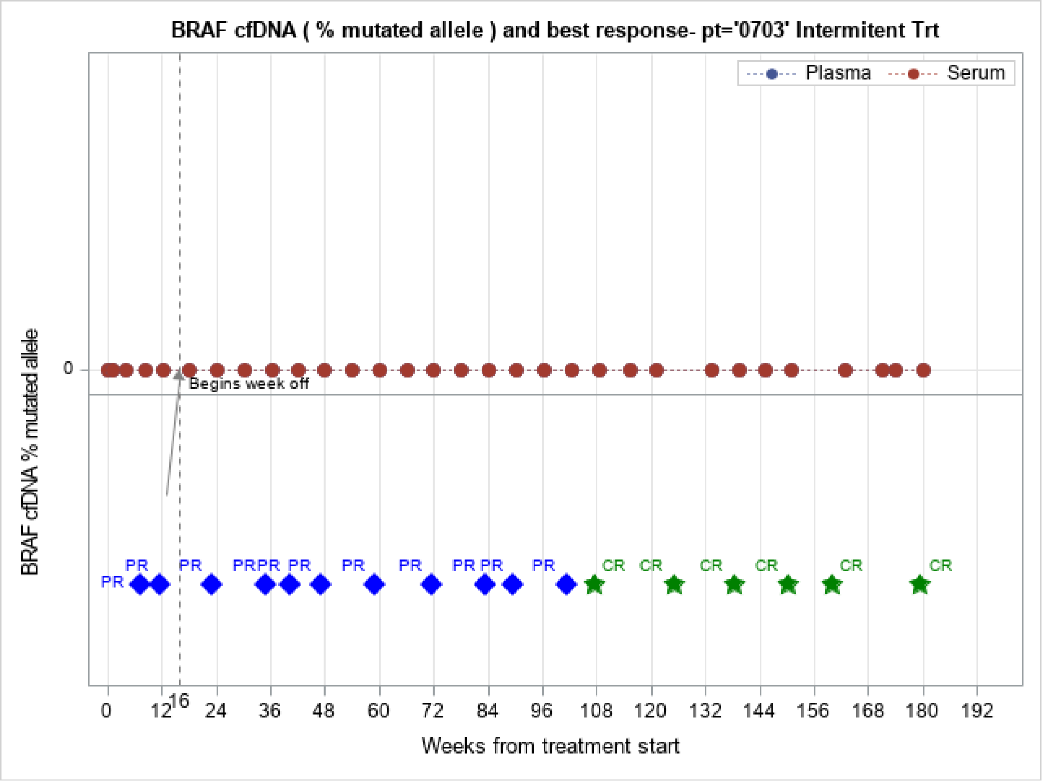

BRAF cfDNA (pg/□L mutated allele) and best response- pt=703 TRT B

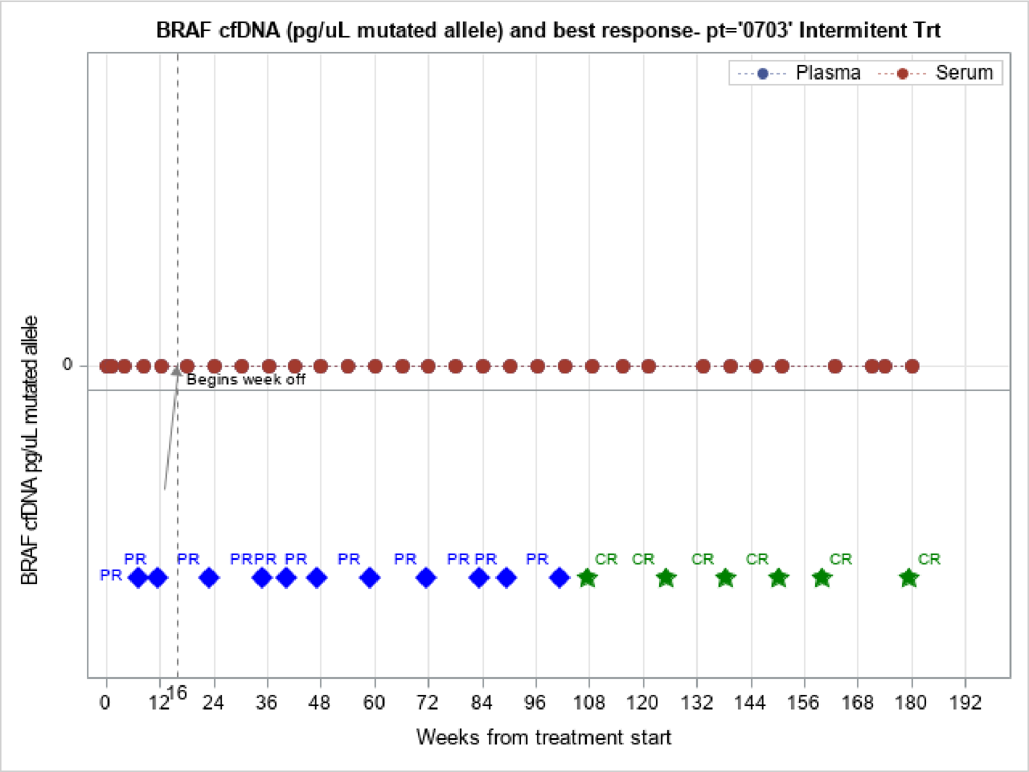

BRAF cfDNA (% mutated allele) and best response- pt=1005 TRT B

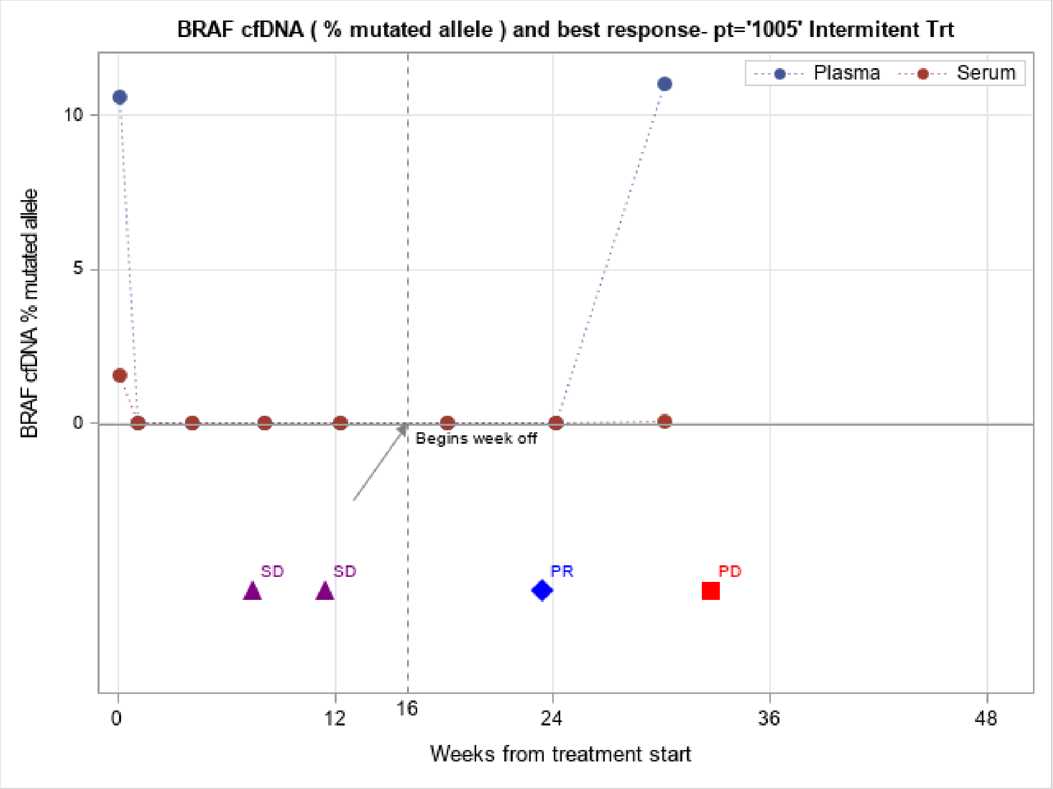

BRAF cfDNA (pg/□L mutated allele) and best response- pt=1005 TRT B

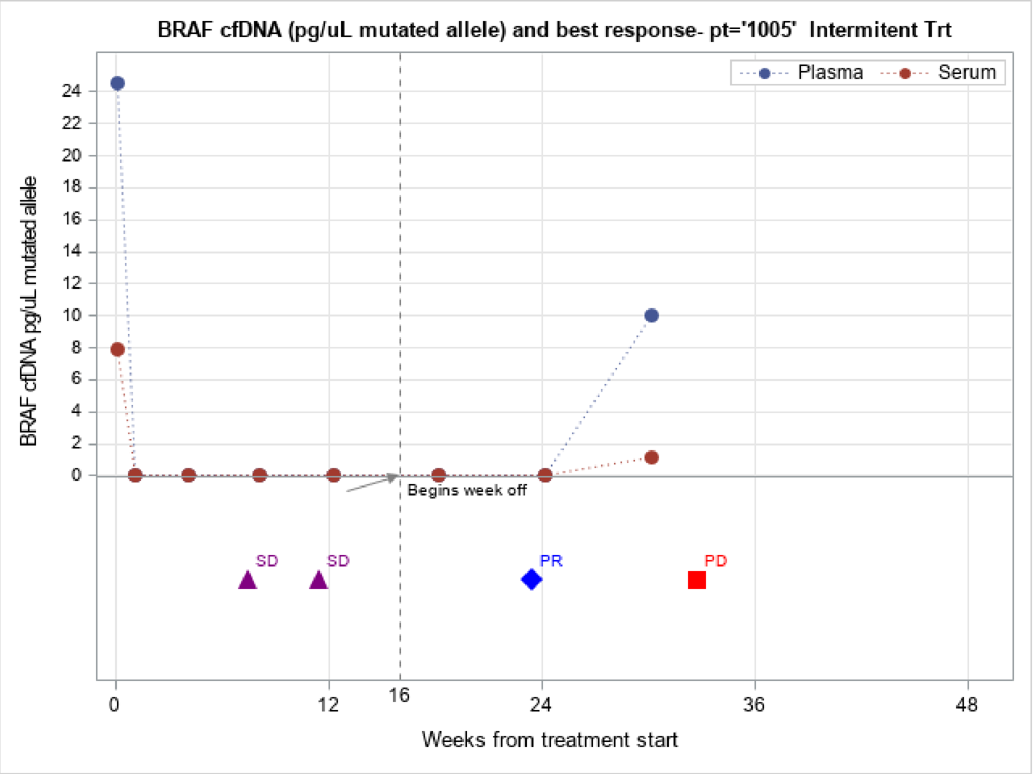

BRAF cfDNA (% mutated allele) and best response- pt=1502 TRT B

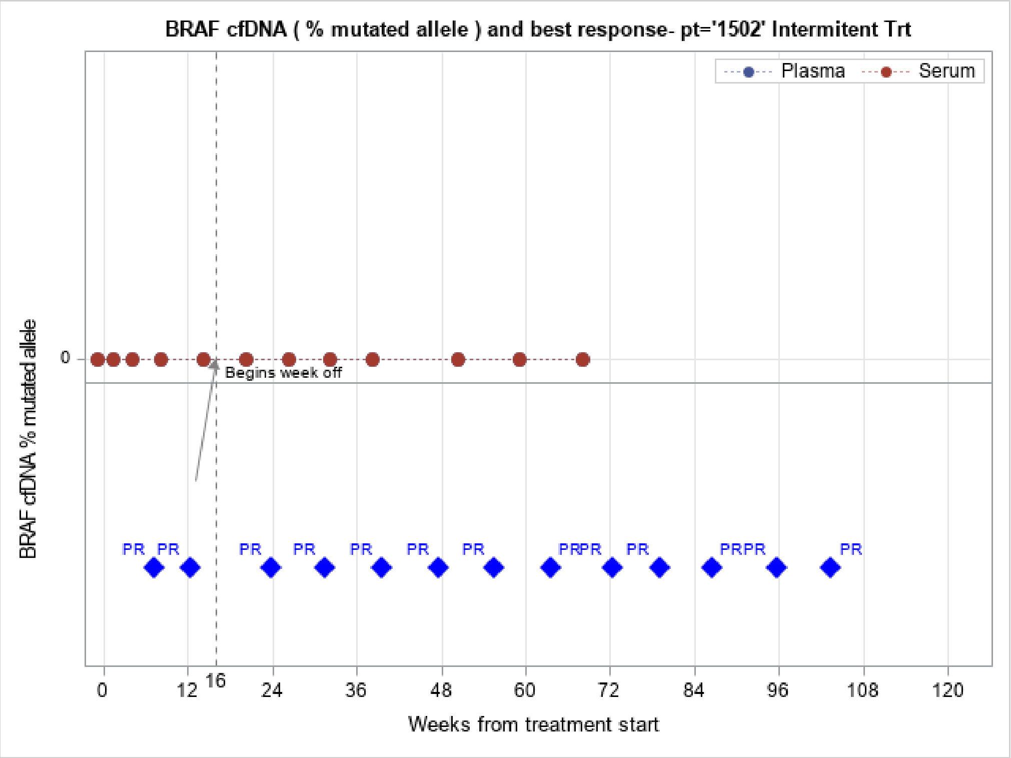

BRAF cfDNA (pg/□L mutated allele) and best response- pt=1502 TRT B

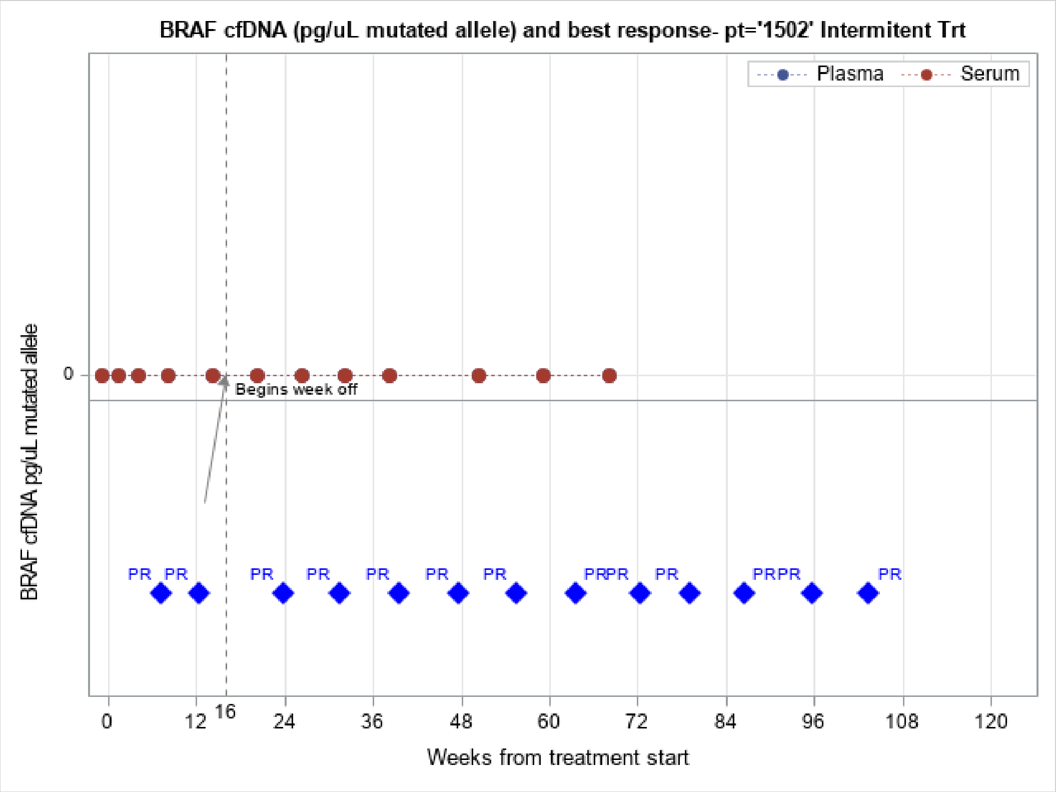

BRAF cfDNA (% mutated allele) and best response- pt=1601 TRT B

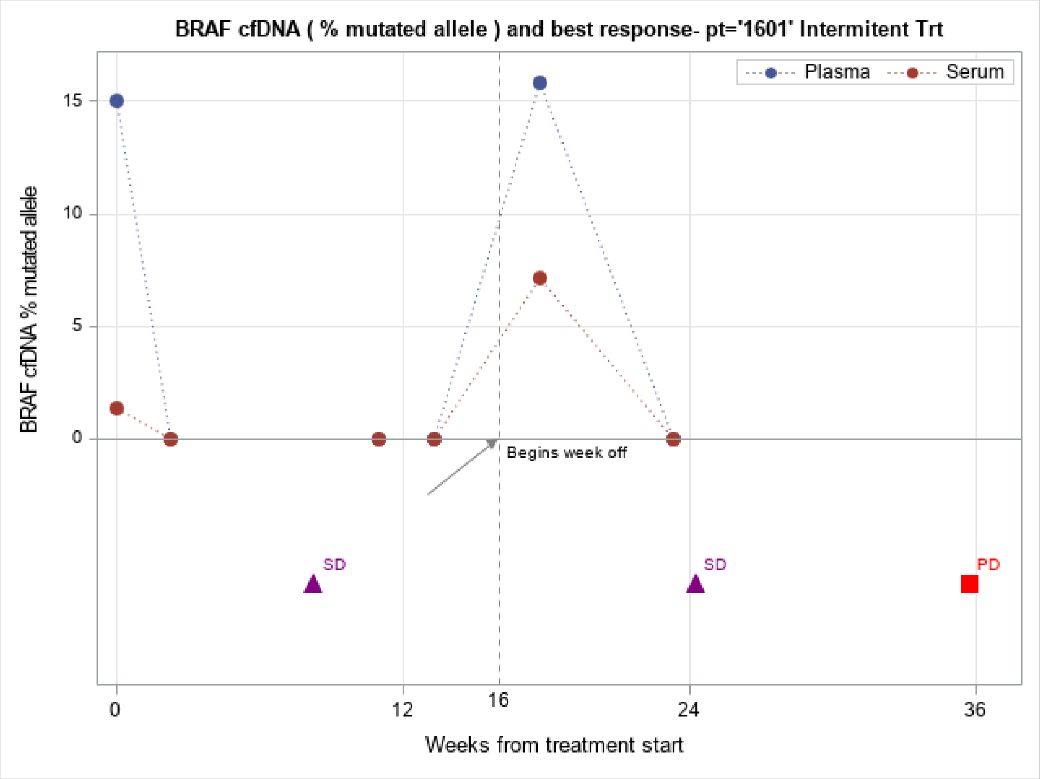

BRAF cfDNA (pg/□L mutated allele) and best response- pt=1601 TRT B

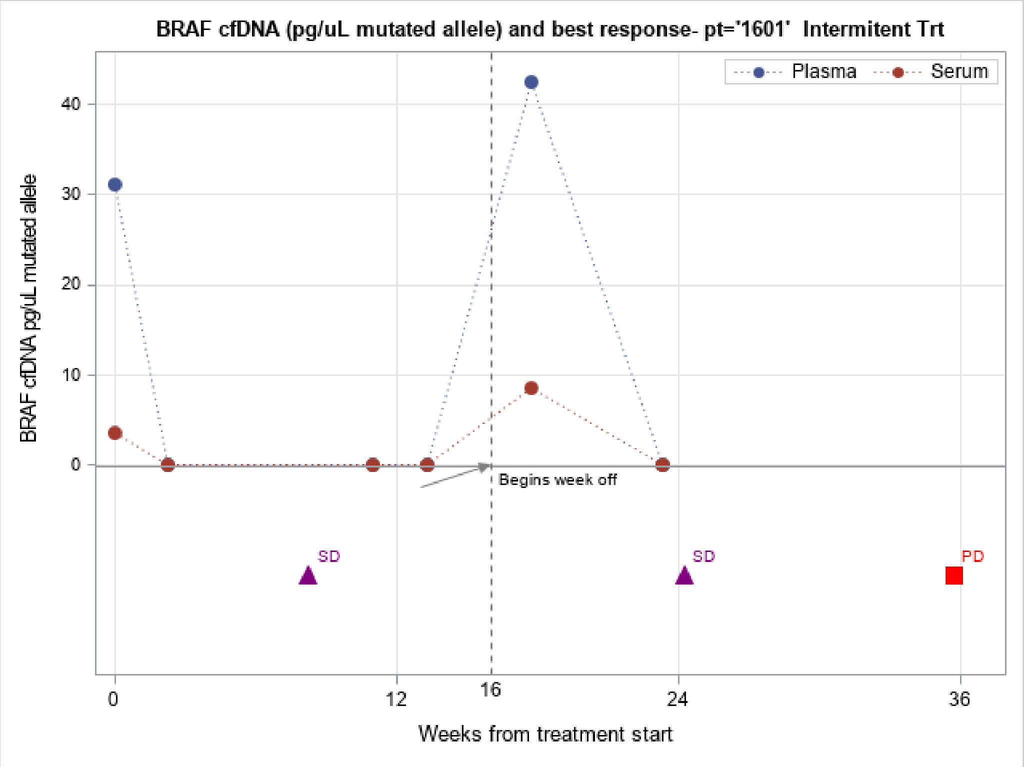

BRAF cfDNA (% mutated allele) and best response- pt=1802 TRT B

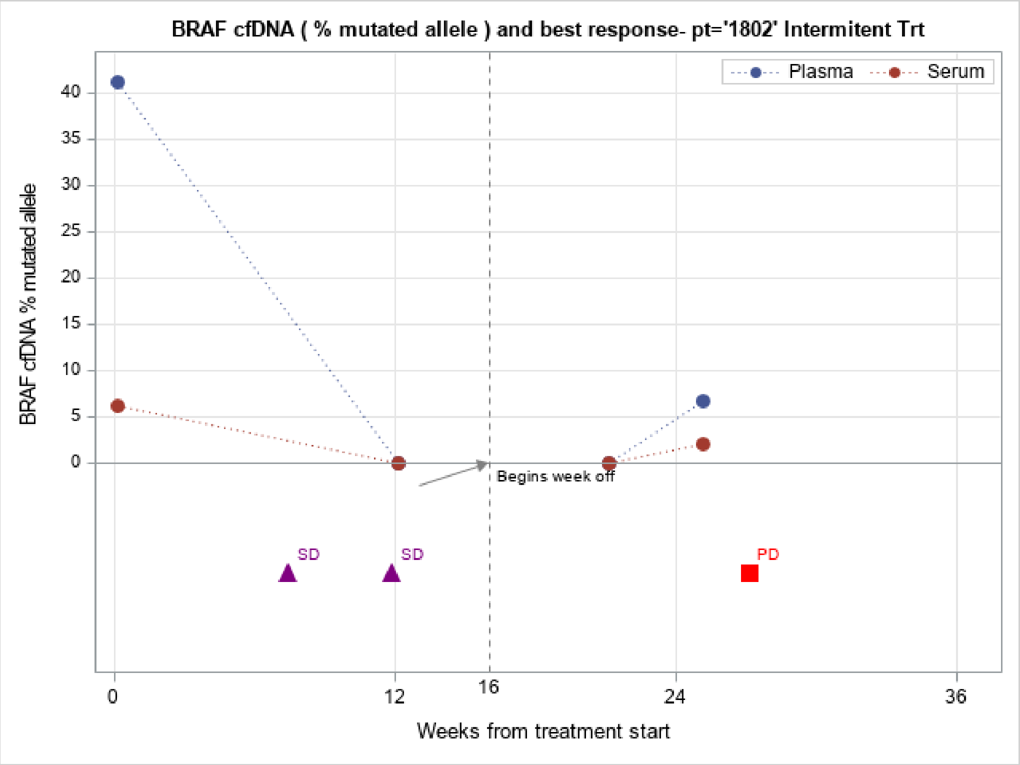

BRAF cfDNA (pg/□L mutated allele) and best response- pt=1802 TRT B

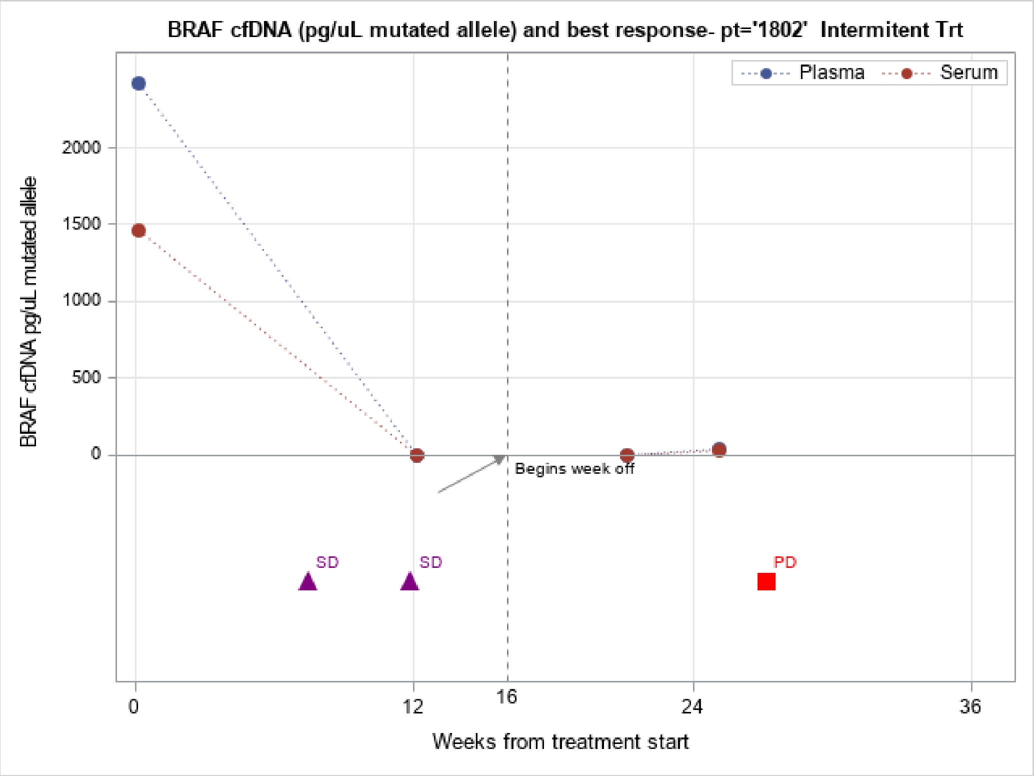

BRAF cfDNA (% mutated allele) and best response- pt=1904 TRT B

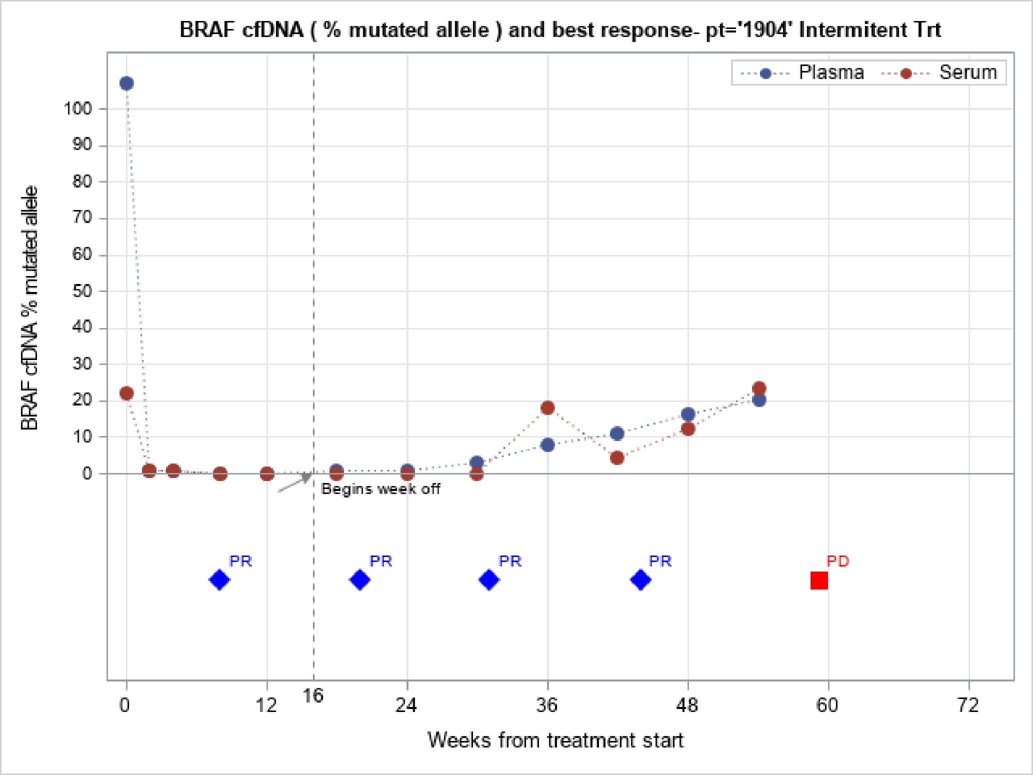

BRAF cfDNA (pg/µL mutated allele) and best response- pt=1904 TRT B

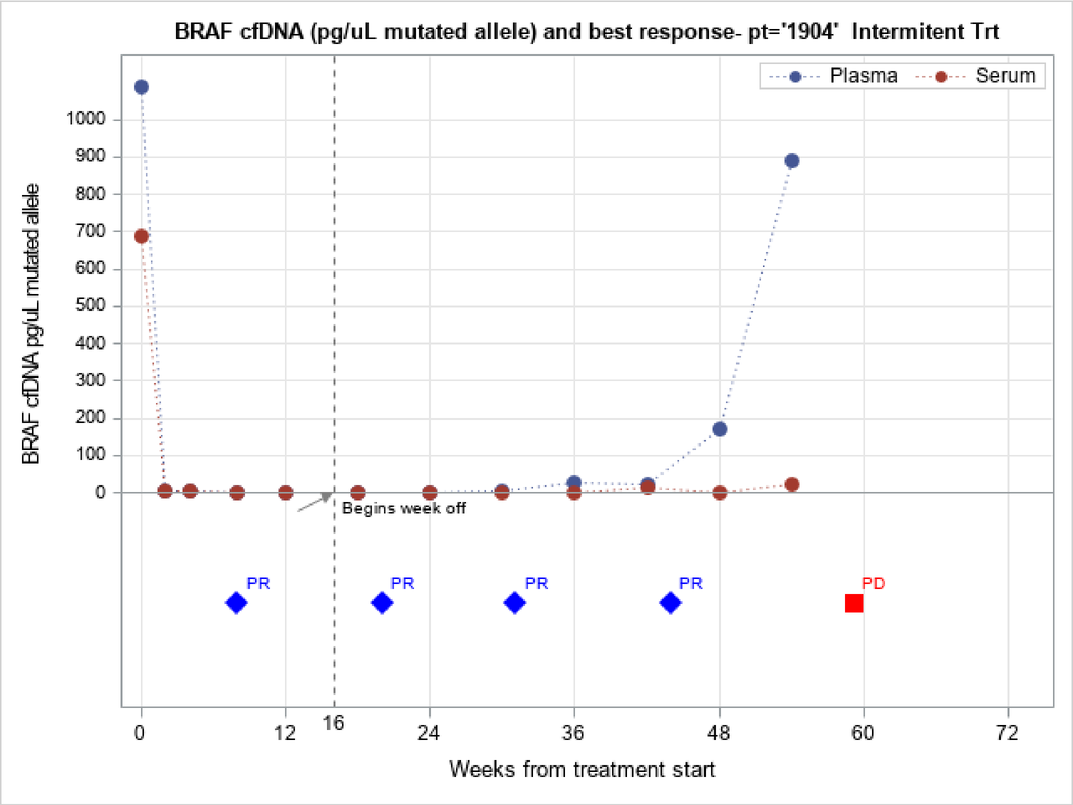

Supplementary Figure 7. Evolution along time of BRAFV600 mutation in cfDNA and tumor response by patient-treatment arm. A (continuous) and B (intermittent). In this section, a graphic of the evolution of BRAF cfDNA in plasma and serum over time and the tumor response is presented for some patients The y axis will correspond to the measures of cfDNA using pg/uL and % of mutated allele . The x axis will show the time, in weeks, from the treatment start date.

Footnote: TRT: Treatment arm

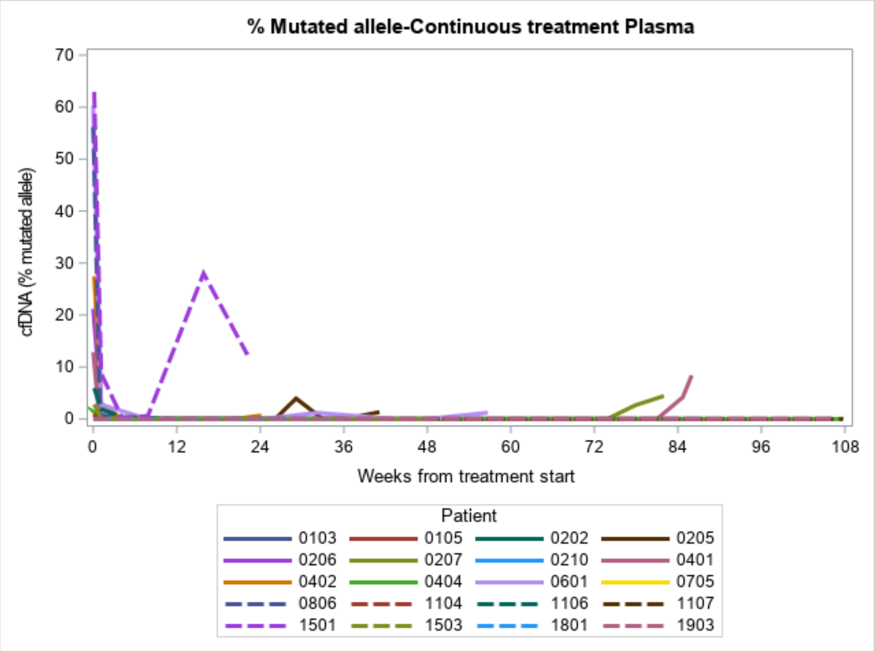

Supplementary Figure 8A. Evolution along time of BRAFV600 mutation in cfDNA in treatment arm A. In this section, a graphic of the evolution of BRAF cfDNA in blood over time and the tumor response is presented for all patients. The y axis will correspond to the measures of cfDNA using % of mutated allele. The x axis will show the time, in weeks, from the treatment start date.

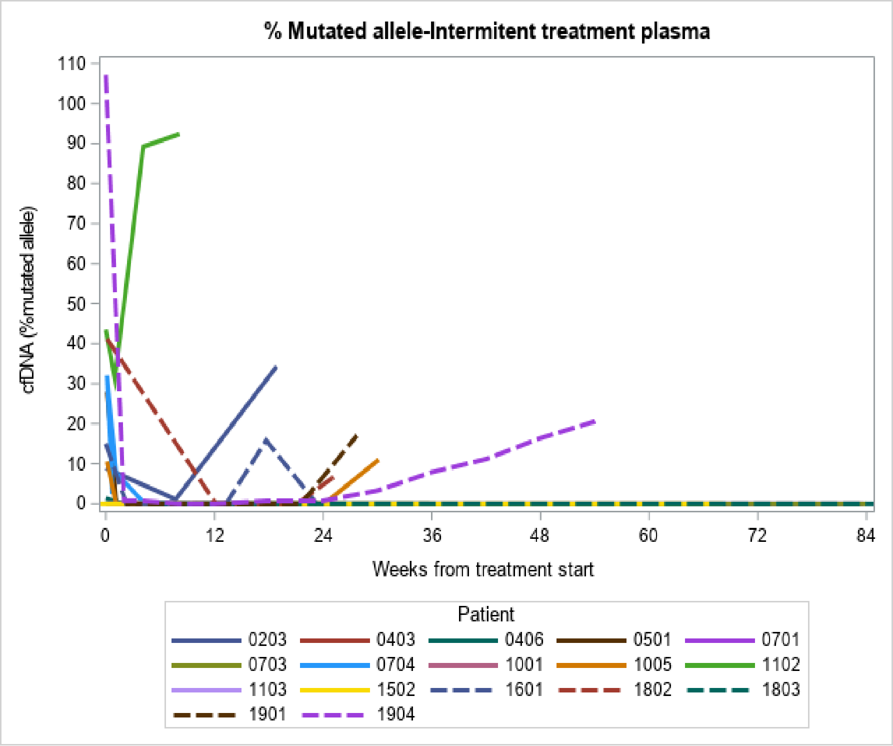

Supplementary Figure 8B. Evolution along time of BRAFV600 mutation in cfDNA in treatment arm B. In this section, a graphic of the evolution of BRAF cfDNA in blood over time and the tumor response is presented for all patients. The y axis will correspond to the measures of cfDNA using % of mutated allele. The x axis will show the time, in weeks, from the treatment start date.
