# Supplementary material for: Intermittent BRAF inhibition in advanced BRAF mutated melanoma results of a phase II randomized trial
Source: Nat Commun. 2021 Dec 1;12:7008. doi: 10.1038/s41467-021-26572-6 (PMC8636498; doi:10.1038/s41467-021-26572-6)
Supplement: Supplementary file 3 — Reporting Summary [file 41467_2021_26572_MOESM3_ESM.pdf]

nature research

Corresponding author(s): M Gonzalez-Cao; A. Berrocal

Last updated by author(s): Jul 11, 2021

Reporting Summary

Nature Research wishes to improve the reproducibility of the work that we publish. This form provides structure for consistency and transparency in reporting. For further information on Nature Research policies, see our Editorial Policies and the Editorial Policy Checklist.

Statistics

For all statistical analyses, confirm that the following items are present in the figure legend, table legend, main text, or Methods section.

n/a	Confirmed	
☐	☒	The exact sample size (*n*) for each experimental group/condition, given as a discrete number and unit of measurement
☐	☒	A statement on whether measurements were taken from distinct samples or whether the same sample was measured repeatedly
☐	☒	The statistical test(s) used AND whether they are one- or two-sided<br>*Only common tests should be described solely by name; describe more complex techniques in the Methods section.*
☐	☒	A description of all covariates tested
☐	☒	A description of any assumptions or corrections, such as tests of normality and adjustment for multiple comparisons
☐	☒	A full description of the statistical parameters including central tendency (e.g. means) or other basic estimates (e.g. regression coefficient) AND variation (e.g. standard deviation) or associated estimates of uncertainty (e.g. confidence intervals)
☐	☒	For null hypothesis testing, the test statistic (e.g. *F*, *t*, *r*) with confidence intervals, effect sizes, degrees of freedom and *P* value noted<br>*Give P values as exact values whenever suitable.*
☒	☐	For Bayesian analysis, information on the choice of priors and Markov chain Monte Carlo settings
☐	☒	For hierarchical and complex designs, identification of the appropriate level for tests and full reporting of outcomes
☐	☒	Estimates of effect sizes (e.g. Cohen's *d*, Pearson's *r*), indicating how they were calculated

Our web collection on statistics for biologists contains articles on many of the points above.

Software and code

Policy information about availability of computer code

Data collection	SAS Software V9.4
Data analysis	Statistical analyses were performed with the use of SAS Software V9.4 QIAGEN Clinical Insight Analyze (QCI-A) software 1.1

For manuscripts utilizing custom algorithms or software that are central to the research but not yet described in published literature, software must be made available to editors and reviewers. We strongly encourage code deposition in a community repository (e.g. GitHub). See the Nature Research guidelines for submitting code & software for further information.

Data

Policy information about availability of data

All manuscripts must include a data availability statement. This statement should provide the following information, where applicable:

- Accession codes, unique identifiers, or web links for publicly available datasets
- A list of figures that have associated raw data
- A description of any restrictions on data availability

Source data are provided with this paper

All data (to replicate every analysis in the manuscript and any Supplementary Information) will be posted to the HARVARD dataverse (https://doi.org/10.7910/DVN/TFFSGR, Harvard Dataverse, DRAFT VERSION). De-identified patient-level data, including the numbers, tables and figures in the paper, will be made available. The protocol and the informed consent form are available in the Supplementary Information

Field-specific reporting

Life sciences study design

All studies must disclose on these points even when the disclosure is negative.

Sample size	Sample size calculation: “Using the method described by Brookmeyer R, for an error α = 0.1 and an error β = 0.20, it will be necessary to include 34 evaluable patients per treatment group, using Log-rank (Mantel-Cox) 2-sided test. With this sample size we would have a power of 80% to detect a difference of 23% in the percentage of patients free of progression to 1 year (with an error α = 0.1)”
Data exclusions	All analysis were performed by intended to treat population (iTTP) No data were excluded
Replication	NA for treatment of patients. Analysis of BRAV600 mutations in cfDNA were performed in quadruplicate: two aliquots of serum and two aliquots of plasma per patient with similar results
Randomization	Patients were randomized to one of the following treatment regimens: Group A (continuous administration) Vemurafenib 960 mg p.o. twice daily on days 1-28 and cobimetinib 60 mg p.o. once a day on days 1-21 of each 28-day treatment cycle. Group B (intermittent administration) Vemurafenib 960 mg p.o. twice daily on days 1-28 and cobimetinib 60 mg p.o. once a day on days 1-21 of each 28-day treatment cycle for 12 weeks. Then, both drugs were administered at the same doses previously indicated, but with an intermittent schedule: Vemurafenib days 1-28 followed by 14 days of rest (4 weeks on and 2 weeks off), and Cobimetinib days 1-21 followed by 21 rest days. (3 weeks on and 3 weeks off).
Blinding	This is a open-label trial because the primary end point was survival and the schedule of administration of treatment in both arms was different

Reporting for specific materials, systems and methods

We require information from authors about some types of materials, experimental systems and methods used in many studies. Here, indicate whether each material, system or method listed is relevant to your study. If you are not sure if a list item applies to your research, read the appropriate section before selecting a response.

Materials & experimental systems

n/a	Involved in the study	
☒	☐	Antibodies
☒	☐	Eukaryotic cell lines
☒	☐	Palaeontology and archaeology
☒	☐	Animals and other organisms
☐	☒	Human research participants
☐	☒	Clinical data
☒	☐	Dual use research of concern

Methods

n/a	Involved in the study	
☒	☐	ChIP-seq
☒	☐	Flow cytometry
☒	☐	MRI-based neuroimaging

Human research participants

Policy information about studies involving human research participants

Population characteristics	Eligible patients were Patients with histologically confirmed melanoma, either unresectable stage IIIc or stage IV metastatic melanoma. Patients must be naïve to treatment for locally advanced unresectable or metastatic disease. Documentation of BRAFV600 mutation-positive status in melanoma tumor tissue. Other inclusion criteria were having measurable disease per RECIST v1.1 and ECOG performance status of 0 or 1. Patient characteristics are included in eTable 1. Median age was 57 years old (range 29 to 85) and 47% were women. Most patients had ECOG 0 (57%), were cutaneous melanomas (81%) and stage M1C (51%)
Recruitment	Before conducting the screening procedures, the investigator obtained written informed consent from each individual participating in this study after the investigator had explained to each subject or guardian/legally authorized representative the nature of the study, the purpose, the procedures involved, the expected duration, the potential risks and benefits involved, any potential discomfort, potential alternative procedure(s) or course(s) of treatment available to the subject, and the extent of maintaining the confidentiality of the subject's records. At the time of enrollment, patients were stratified with a 1: 1 ratio according to: ECOG functional status (PS 0 vs 1). LDH levels (normal vs elevated), age (65 years or older) and staging (IIIc or IV). Patients were not randomized according to the number of metastatic sites, so an imbalance between arms, in arm A 37% of patients had melanoma in more than 2 sites and in arm B 51% had more than two affected sites, could have an impact in survival results
Ethics oversight	Each subject was informed that participation in the study was voluntary. The protocol, informed consent forms (ICF), and any appropriate related documents were submitted to the Institutional Review Board (IRB) or Independent Ethics Committee (IEC) by the principal investigator (PI) for approval.

Note that full information on the approval of the study protocol must also be provided in the manuscript.

Clinical data

Policy information about clinical studies

All manuscripts should comply with the ICMJE guidelines for publication of clinical research and a completed CONSORT checklist must be included with all submissions.

Clinical trial registration	EUDRA CT Number 2014-005277-36; NCT02583516
Study protocol	The protocol (including the statistical analysis plan) and the informed consent form are available in the Supplementary Information

Data collection	Data collection from june 2015 to September 2019
Outcomes	Progression-free survival and overall survival were estimated by means of the Kaplan–Meier method and the nonparametric log-rank test was applied for comparisons of groups. Cox semiparametric proportional-hazards model was used in the analysis of survival data to explain the effect of explanatory variables on hazard rates, obtaining Hazard Ratios (HR) and their 95% confidence intervals (CI). The descriptive statistics include mean, standard deviation, median, range for continuous variables and the number and percentages for categorical variables. Association analysis used the Fisher exact test or Chi-square test for categorical variables. In case of continuous variables t-test Anova or the non-parametric Wilcoxon (Mann-Whitney), as applicable.<br>Exploratory analysis of the relation between cfDNA BRAF expression values and treatment response during the study was achieved with graphical display of results. Each analysis was performed with the use of a two-sided 5% significance level and a 95% CI.<br>Place were clinical trail was performed were: 1. Translational Cancer Research Unit, Instituto Oncologico Dr Rosell, Dexeus University Hospital, Barcelona, Spain; 2. Hospital Universitario de Canarias, Tenerife, Spain; 3. Hospitales Universitarios Regional y Virgen de la Victoria (HURyVV), Málaga, Spain; 4. Hospital Universitario Virgen Macarena, Sevilla, Spain; 5. Hospital Clínico Universitario Virgen de la Arrixaca, Murcia, Spain; 6. Hospital Clinic, Barcelona, Spain; 7. Hospital Valle Hebron, Barcelona, Spain; 8. Hospital Universitario la Paz, Madrid, Spain; 9. Hospital Miguel Servet, Zaragoza, Spain; 10. Hospital Universitario la Fe, Valencia, Spain; 11. Hospital Universitario Dr Peset, Valencia, Spain; 12. Hospital de Vigo, Pontevedra, Spain; 13. Hospital Universitario de Donostia, Guipuzkoa, Spain; 14. Hospital Universitario de Salamanca, Salamanca, Spain; 15. Hospital Insular Las Palmas, Las Palmas de Gran Canaria, Spain; 16.Hospital Lucus Agusti, Lugo, Spain; 17. Hospital del Mar, Barcelona, Spain; 18. Hospital 12 de Octubre, Madrid, Spain; 19. Hospital General de Valencia, Valencia, Spain
