# Supplementary material for: Intermittent BRAF inhibition in advanced BRAF mutated melanoma results of a phase II randomized trial
Source: Nat Commun. 2021 Dec 1;12:7008. doi: 10.1038/s41467-021-26572-6 (PMC8636498; doi:10.1038/s41467-021-26572-6)
Supplement: Supplementary file 4 — Source Data [file 41467_2021_26572_MOESM4_ESM.zip › Contents of GEM0511 sent datasets(09JUL21).rtf]

Contents of gem0511_survival.xlsx

The CONTENTS Procedure	

Data Set Name	GEM0511_SURVIVAL	Observations	70	
Member Type	DATA	Variables	7	
Engine	V9	Indexes	0	
Created	09/07/2021 06:42:53	Observation Length	72	
Last Modified	09/07/2021 06:42:53	Deleted Observations	0	
Protection		Compressed	NO	
Data Set Type		Sorted	NO	
Label				
Data Representation	WINDOWS_64			
Encoding	wlatin1  Western (Windows)			


Engine/Host Dependent Information	
Data Set Page Size	65536	
Number of Data Set Pages	1	
First Data Page	1	
Max Obs per Page	908	
Obs in First Data Page	70	
Number of Data Set Repairs	0	
ExtendObsCounter	YES	
Filename	C:\Users\Ana\AppData\Local\Temp\SAS Temporary Files\_TD18172_DESKTOP-V2D45KF_\survival_tosend.sas7bdat	
Release Created	9.0401M5	
Host Created	X64_10PRO	
Owner Name	DESKTOP-V2D45KF\Ana	
File Size	128KB	
File Size (bytes)	131072	


Variables in Creation Order	
#	Variable	Type	Len	
1	arm	Char	20	
2	gstage	Char	10	
3	cspfs	Num	8	
4	pfsm	Num	8	
5	osm	Num	8	
6	csos	Num	8	
7	subjid	Num	8	


Contents of Gem0511_survival_SS.xlsx

The CONTENTS Procedure	

Data Set Name	GEM0511_SURVIVAL_SS	Observations	34	
Member Type	DATA	Variables	8	
Engine	V9	Indexes	0	
Created	09/07/2021 09:27:54	Observation Length	72	
Last Modified	09/07/2021 09:27:54	Deleted Observations	0	
Protection		Compressed	NO	
Data Set Type		Sorted	NO	
Label				
Data Representation	WINDOWS_64			
Encoding	wlatin1  Western (Windows)			


Engine/Host Dependent Information	
Data Set Page Size	65536	
Number of Data Set Pages	1	
First Data Page	1	
Max Obs per Page	908	
Obs in First Data Page	34	
Number of Data Set Repairs	0	
ExtendObsCounter	YES	
Filename	C:\Users\Ana\AppData\Local\Temp\SAS Temporary Files\_TD18172_DESKTOP-V2D45KF_\surv_ss_tosend.sas7bdat	
Release Created	9.0401M5	
Host Created	X64_10PRO	
Owner Name	DESKTOP-V2D45KF\Ana	
File Size	128KB	
File Size (bytes)	131072	


Variables in Creation Order	
#	Variable	Type	Len	Format	Informat	Label	
1	arm	Char	20				
2	cspfs	Num	8				
3	pfsm	Num	8				
4	osm	Num	8				
5	csos	Num	8				
6	LBGYN	Char	8	$8.	$8.	Generic YN - Lab Test Results	
7	g	Char	1				
8	subjid	Num	8				


Contents of Gem0511_BRAF_LDH_SS.xlsx

The CONTENTS Procedure	

Data Set Name	GEM0511_BRAF_LDH_SS	Observations	33	
Member Type	DATA	Variables	7	
Engine	V9	Indexes	0	
Created	09/07/2021 07:31:54	Observation Length	80	
Last Modified	09/07/2021 07:31:54	Deleted Observations	0	
Protection		Compressed	NO	
Data Set Type		Sorted	NO	
Label				
Data Representation	WINDOWS_64			
Encoding	wlatin1  Western (Windows)			


Engine/Host Dependent Information	
Data Set Page Size	65536	
Number of Data Set Pages	1	
First Data Page	1	
Max Obs per Page	817	
Obs in First Data Page	33	
Number of Data Set Repairs	0	
ExtendObsCounter	YES	
Filename	C:\Users\Ana\AppData\Local\Temp\SAS Temporary Files\_TD18172_DESKTOP-V2D45KF_\braf_ldh_tosend.sas7bdat	
Release Created	9.0401M5	
Host Created	X64_10PRO	
Owner Name	DESKTOP-V2D45KF\Ana	
File Size	128KB	
File Size (bytes)	131072	


Variables in Creation Order	
#	Variable	Type	Len	
1	ARM	Char	20	
2	GLDHBRAF1	Char	20	
3	cspfs	Num	8	
4	pfsm	Num	8	
5	osm	Num	8	
6	csos	Num	8	
7	SUBJID	Num	8	


Contents of gem0511_braf_resp_pct.xlsx

The CONTENTS Procedure	

Data Set Name	GEM0511_.BRAF_RESP_PCT	Observations	1350	
Member Type	DATA	Variables	15	
Engine	V9	Indexes	0	
Created	09/07/2021 07:49:21	Observation Length	240	
Last Modified	09/07/2021 07:49:21	Deleted Observations	0	
Protection		Compressed	NO	
Data Set Type		Sorted	NO	
Label				
Data Representation	WINDOWS_64			
Encoding	wlatin1  Western (Windows)			


Engine/Host Dependent Information	
Data Set Page Size	65536	
Number of Data Set Pages	6	
First Data Page	1	
Max Obs per Page	272	
Obs in First Data Page	261	
Number of Data Set Repairs	0	
ExtendObsCounter	YES	
Filename	C:\Users\Ana\AppData\Local\Temp\SAS Temporary Files\_TD18172_DESKTOP-V2D45KF_\braf_resp_pct_tosend.sas7bdat	
Release Created	9.0401M5	
Host Created	X64_10PRO	
Owner Name	DESKTOP-V2D45KF\Ana	
File Size	448KB	
File Size (bytes)	458752	


Variables in Creation Order	
#	Variable	Type	Len	Format	Informat	Label	
1	trt	Char	40				
2	lresp	Char	30	$RESPENG.	$30.	Respuesta Global	
3	PT	Char	10	$10.	$10.	Patient	
4	ciclo	Num	8	BEST12.	F12.		
5	timeto_assw	Num	8				
6	BRAFDAT	Num	8	DDMMYY10.			
7	LBSPEC	Char	40	$40.	$40.	Specimen Type	
8	LBTEST	Char	40	$40.	$40.	Lab Test or Examination Name	
9	LBGRC	Num	8	F10.3	F10.	Original numeric result (nonCD)	
10	TIMETOW	Num	8			Time from treatment start(weeks)	
11	tiempomax	Num	8				
12	ypos	Num	8				
13	DSPERF	Char	2	$2.	$2.	Participate in substudy?	
14	NUMERO	Num	8				
15	SUBJID	Num	8				
